# Chemical Analysis of Exhaled Vape Emissions: Unraveling the Complexities of Humectant Fragmentation in a Human Trial Study

**DOI:** 10.1021/acs.chemrestox.4c00088

**Published:** 2024-05-21

**Authors:** Katherine S. Hopstock, Véronique Perraud, Avery B. Dalton, Barbara Barletta, Simone Meinardi, Robert M. Weltman, Megan A. Mirkhanian, Krisztina J. Rakosi, Donald R. Blake, Rufus D. Edwards, Sergey A. Nizkorodov

**Affiliations:** † Department of Chemistry, University of California, Irvine, California 92697, United States; ‡ Program in Public Health, University of California, Irvine, California 92697, United States

## Abstract

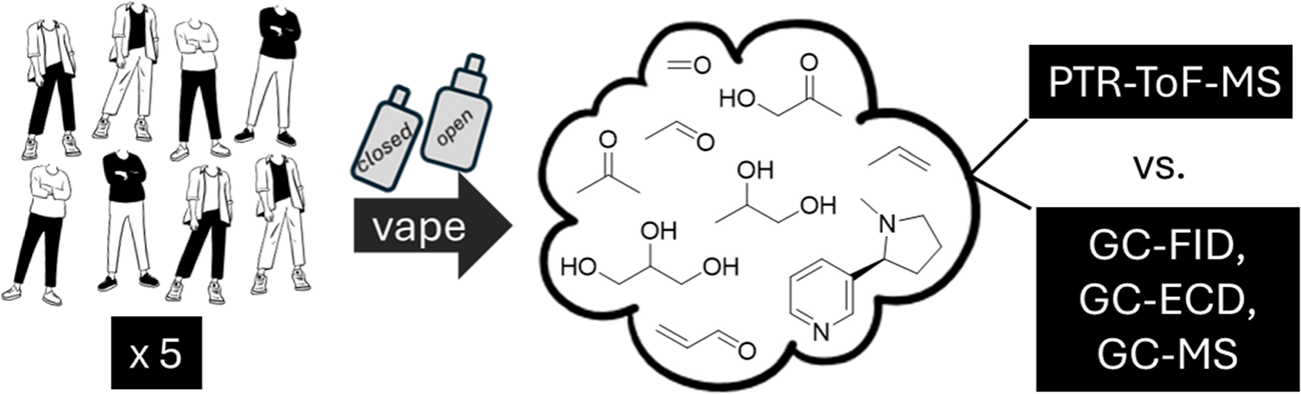

Electronic cigarette smoking (or vaping) is on the rise, presenting questions about the effects of secondhand exposure. The chemical composition of vape emissions was examined in the exhaled breath of eight human volunteers with the high chemical specificity of complementary online and offline techniques. Our study is the first to take multiple exhaled puff measurements from human participants and compare volatile organic compound (VOC) concentrations between two commonly used methods, proton-transfer-reaction time-of-flight mass spectrometry (PTR-ToF-MS) and gas chromatography (GC). Five flavor profile groups were selected for this study, but flavor compounds were not observed as the main contributors to the PTR-ToF-MS signal. Instead, the PTR-ToF-MS mass spectra were overwhelmed by e-liquid thermal decomposition and fragmentation products, which masked other observations regarding flavorings and other potentially toxic species associated with secondhand vape exposure. Compared to the PTR-ToF-MS, GC measurements reported significantly different VOC concentrations, usually below those from PTR-ToF-MS. Consequently, PTR-ToF-MS mass spectra should be interpreted with caution when reporting *quantitative* results in vaping studies, such as doses of inhaled VOCs. Nevertheless, the online PTR-ToF-MS analysis can provide valuable *qualitative* information by comparing relative VOCs in back-to-back trials. For example, by comparing the mass spectra of exhaled air with those of direct puffs, we can conclude that harmful VOCs present in the vape emissions are largely absorbed by the participants, including large fractions of nicotine.

## Introduction

Electronic cigarettes (e-cigarettes, also known as vapes) are battery-powered devices that convert an e-liquid into an aerosol that can be inhaled by an individual. Since these devices deliver nicotine without the combustion of tobacco, vapes are often marketed as a safer and healthier alternative to traditional cigarettes.^1^ Modern vape device usage is perceived to be a more socially acceptable alternative to traditional combustible cigarettes, giving rise to users discretely vaping in public spaces where smoking is normally prohibited.^2^ In addition, fruit and candy flavors are appealing to a younger demographic, posing new risks of nicotine addiction in developing youth, as 10% of middle and high school students reported vape usage in 2023.^3−6^ The long-term effects of vaping are unknown, but research has demonstrated that flavoring agents can induce inflammation, endothelial dysfunction, epithelial barrier disruption, oxidative stress, DNA damage, electrophysiological alterations, immunomodulatory effects, and behavioral changes, even independent of nicotine.^7^ There is thus a need for a more comprehensive physiochemical characterization of vaping emissions, and more studies that can link emission properties to specific toxicological outcomes.^8^

Most vape devices consist of a lithium battery, a heating element, a liquid tank/cartridge, and e-liquid.^9,10^ Deviations in the design are determined by the class of vape device, subsequently referred to as “*closed*” and “*open*” in this text. *Closed* devices are preloaded, disposable devices that are not intended to be refilled or have their battery/atomizers replaced by the user.^11^*Open* devices are tank-based systems that are intended to have the e-liquid refilled, parts replaced, and power outputs manipulated.^11^ These devices are often larger in size than *closed* devices and have higher-voltage batteries and/or lower-resistance heating elements that can produce a more concentrated smoke than *closed* devices.

Together, the battery voltage and resistance of the heating element (coils) determine the e-cigarette power output. This, in turn, directly impacts the concentration of aerosol to be inhaled by an individual.^12^ E-liquid is composed of three main ingredients: nicotine (although some e-liquids are nicotine-free), humectants (to prevent the e-liquid from drying out), and flavoring agents.^11^ The humectant component is typically a mixture of propylene glycol (PG; C_3_H_8_O_2_, MW 76.095 g mol^–1^) and glycerol (GLY; C_3_H_8_O_3_, MW 92.09 g mol^–1^). These are considered safe for human use, however, the literature suggests that dangerous thermal degradation products are inhaled during vaping, as these species are produced when coil temperatures exceed 130 °C.^11,13−17^ These include glycols, aldehydes, polycyclic aromatic hydrocarbons (PAHs), and other volatile organic compounds (VOCs). In indoor environments, individuals in the vicinity of vape users may be exposed to these VOCs through unintentional, secondhand inhalation.^18^

Extensive research has utilized offline analytical techniques which include gas and liquid chromatography (GC-MS/FID, LC-MS), often coupled to derivatization methods prior to analysis (e.g., 1,4-dinitrophenylhydrazine, DNPH), to assess the presence of VOCs in vape emissions.^19−26^ The proton-transfer-reaction time-of-flight mass spectrometry (PTR-ToF-MS), an online technique, has proven advantageous in online vaping studies as it provides fast-response measurements of VOCs without additional sample treatment or preparation. The PTR-ToF-MS has been previously applied to exhaled breath analysis,^27−35^ but only a few studies have utilized this online monitoring technique for direct vape emissions, and even fewer studies have utilized this instrument to examine the exhaled vape emissions from human participants. For example, Blair et al. (2015) demonstrated proof-of-concept that the PTR-ToF-MS could be used to quantify VOC concentrations in direct e-cigarette emissions.^36^ This study only reported the concentrations of five VOCs (acetaldehyde, acetone, acetonitrile, acrolein, and methanol) from the analysis of one vape device, independent of human participants.^36^ O’Connell et al. (2015) used a PTR-ToF-MS to determine exhaled nicotine concentrations (1.8–1786 ppb) of three human volunteers.^37^ Breiev et al. (2016) calibrated the PTR-ToF-MS for PG, GLY, and nicotine then conducted proof-of-concept sampling with one human participant.^38^ Through the implementation of a dilution setup, they found agreement between measurements taken online (PTR-ToF-MS) and offline (GC-FID) for three VOCs of interest.^38^ Sangani et al. (2021) utilized the PTR-ToF-MS to examine the direct emissions from vape devices used by patients admitted to a hospital for lung injury, but they did not examine the subsequent exhaled breath.^39^ Formaldehyde, acetaldehyde, acetone, PG, cyclohexane, and nicotine were present in the direct emissions from the vaping devices and total VOC concentrations were reported up to 600 ppm.^39^

The present work sought to examine the chemical composition of vape emissions with the PTR-ToF-MS and compare these results to the exhaled breath of eight human volunteers vaping five different flavor classes. To tease out fragmentation induced by the PTR ionization, measurements with an offline GC method were also conducted. Our study is the first to implement a flavor study with human participants and analyze breath samples with the high chemical specificity of complementary online and offline techniques.

## Materials and Methods

### Human Participants, Vape Device Selections, and Sampling Protocols

In January-June 2022, eight individuals each made five separate visits to UC Irvine to participate in a human trial vaping study. This study was approved by the University of California, Irvine Institutional Review Board (UCI IRB # 20216747). All samples were obtained with the informed consent of the human participants. Participants were eligible to participate if they were greater than 21 years of age and had been exclusively vaping (i.e., did not smoke cigarettes) for more than a year at the time of sampling. Each participant was informed of experimental protocols and signed consent forms prior to sample collection. To avoid bias in VOC profiles from prior vaping and/or other metabolic influences, volunteers were required to have not vaped, eaten, or drank anything (other than water) prior to the morning visit.^40−42^ In addition, background samples of laboratory room air and the participant’s exhaled baseline breath were collected (prior to the first vape puff) to determine the incremental increase in exhaled compounds as a result of vaping.

During each visit, the participant used a brand-new vape of the same model/brand habitually used. Participants exhaled vape puffs into 2 L whole air sampling (WAS) canisters and a Tedlar bag for analysis. Inhaled breath topography information (e.g., puff volume, puff duration, puff inhalation rate) was recorded using a Sodium SPA-M analyzer (Körber Technologies). Inhalation volumes and rates were utilized for the PTR-ToF-MS direct injection measurements and calculations (*described below*). Five different flavor profiles were selected for this study—mint, watermelon (or tobacco for participants 2 and 3), apple, vanilla, and mango. Only one flavor was smoked during each visit and flavors were rotated until all five had been smoked by each individual. Tobacco was initially one of the five profiles selected for this study, but due to negative feedback from participants 2 and 3, it was discontinued and switched out for watermelon. Participant 8 smoked a tobacco cream-flavored vape as this was the manufacturer’s alternative for vanilla, and this trial was grouped with all other vanilla trials for analysis. The various e-cigarette devices, settings, flavors, and puff topography information are reported in Table S1.

**Figure 1:**
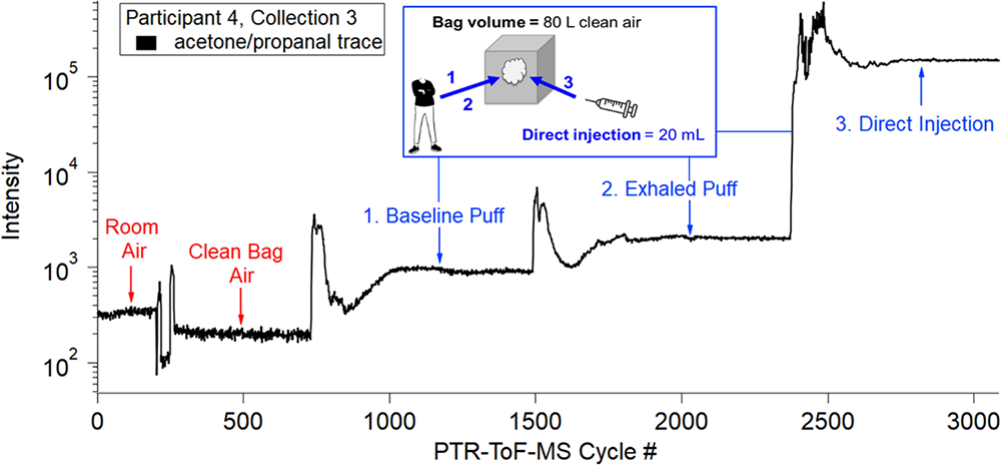
Example of sampling progression using the PTR-ToF-MS for participant 4, collection 3. The acetone/propanal (*m*/*z* 59.0491, C_3_H_7_O^+^) trace was selected (black trace), and the intensity is shown as a function of the PTR-ToF-MS cycle number (each cycle is 1 s). After sampling room air and clean air, a participant exhaled a baseline puff (segment 1) into the bag and the ion trace increased. Once the trace plateaued, the participant inhaled from the vape device and exhaled into the bag (segment 2). Finally, a syringe was directly connected to the vape device and 20 mL of vape aerosol was collected then subsequently pushed into the bag using a syringe pump (segment 3).

Figure 1 shows the sampling progression for an example participant trial (participant 4, collection 3). During each experiment, a Tedlar 100 L air sample bag (Dupont de Nemours) was connected to the PTR-ToF-MS for participant sampling. The bag was initially filled with 80 L of clean dry air prior to each participant sampling visit. Further details on the Tedlar bag treatment can be found in SI Appendix A. PTR-ToF-MS measurements began with 10 min of sampling laboratory room air. The PTR-ToF-MS inlet was then connected to the Tedlar bag and 10 min of clean air sampling was conducted. Using a mouthpiece made of Teflon tubing, the participant exhaled a baseline breath into the bag (no vape). This and subsequent puffs created a spike in the signal because the PTR-ToF-MS sampling port was close to the injection port, but the signal stabilized after several minutes. After the background samples were collected, volunteers were requested to remain sedentary, as physiological changes can induce concentration fluctuations in exhaled breath.^43^ During the PTR-ToF-MS stabilization time, the participant exhaled a baseline breath into a WAS canister. Next, the participant inhaled from an e-cigarette and exhaled into the Tedlar bag. While the PTR-ToF-MS was stabilizing after the exhaled vape puff, the participant inhaled from the vape device again and exhaled into a separate WAS canister. Lastly, a custom-built, programmable syringe pump was used to pull 20 mL of e-cigarette aerosol (directly from each vape device) and inject the aerosol into the Tedlar bag at a rate of 9.3 mL s^–1^ (Appendix A). Direct injection measurements were not collected in WAS canisters for GC analysis due to oversaturating detector responses.

### Online PTR-ToF-MS Measurements

A PTR-ToF-MS (model 8000, Ionicon Analytik GmbH, Innsbruck, Austria) was utilized for the online, real-time analysis of VOCs exhaled by participants without any sample aging or pretreatment. PTR-ToF-MS technology is described in detail by Jordan et al. (2009) and Yuan et al. (2017) and is briefly discussed in the SI, as it pertains to concentration determinations.^44,45^ All PTR-ToF-MS experimental settings are described in SI Appendix A.

Data analysis was conducted using the PTR-MS Viewer 3.4.2 software (Ionicon). Reported VOCs were expected to form [M + H]^+^ ions. However, the observed mass spectra were strongly affected by in-source ion fragmentation and other ion–molecule ionization pathways as discussed below. The PTR-ToF-MS traces were defined as room air, clean air (clean air inside Tedlar bags), background breath (no vape), exhaled puff (with vape), and direct injection of vape aerosol. Data from Pagonis et al. (2019) were especially useful for the identification of compounds detected by the PTR-ToF-MS (Table S4).^46^ The high mass resolving power of PTR-ToF-MS was essential for identifying compounds with the same nominal *m*/*z* values. Figure S2 illustrates the advantage of the high resolving power for three separate samples (exhaled breath, direct injection, and heated glycerol) at nominal masses 43 and 57. Clear separation and resolution of peaks is achieved for *m*/*z* 43.018 (C_2_H_3_O^+^) and 43.054 (C_3_H_7_^+^), as well as at *m*/*z* 57.033 (C_3_H_5_O^+^) and 57.070 (C_4_H_9_^+^). Despite the high resolving power, peaks with the same nominal mass could not be cleanly resolved if they had vastly different abundances. For example, separation could not be achieved at nominal mass 93 (Figure S2) due to an overwhelming signal at *m*/*z* 93.055 (C_3_H_9_O_3_^+^) from humectant glycerol, which made it difficult to differentiate and quantify the much smaller signal from toluene at *m*/*z* 93.070 (C_7_H_9_^+^). Therefore, the signal at *m*/*z* 93 represents a combination of mostly glycerol and some toluene. For compounds observed to increase in comparison to the background breath, concentrations were determined by the PTR-MS Viewer software. Concentration values (ppbv) were converted into mass concentrations (μg VOC puff^–1^) without correcting for various fragmentation pathways. Baseline breath values were subtracted from exhaled breath values. Further details on data analysis can be found in SI Appendix A.

### Whole Air Sample (WAS) Canisters Collection and Offline Gas Chromatography Analysis

Exhaled breath was also collected using evacuated 2 L electropolished whole air sampling (WAS) stainless steel canisters. For each exhaled breath sample, the participant exhaled through a 1/4 in. OD Teflon tube directly into the canister, without any further dilution. After collection, each canister was analyzed within a few days. The gas chromatography (GC) platform used for the quantitative detection of VOCs uses a set of three GCs coupled to various detectors, including a mass spectrometer (GC-MS), flame-induced detector (GC-FID), and an electron capture detector (GC-ECD), working in parallel for the detection of a wide variety of species including alkanes (C2–C10), cycloalkanes (C4–C7), alkenes (C2–C10), ethyne, aromatics (C6–C9), halocarbons (C1–C2), alkyl nitrates (C1–C5), selected sulfur compounds, and selected oxygenated VOCs. A detailed description of the GC platform and analytical procedures for VOC analysis are provided in Colman et al. (2001) and Simpson et al. (2010).^47,48^ WAS canister preparation and GC experimental settings specific to this study are described in SI Appendix B.

The chromatograms were digitally acquired with Chromeleon (version 6.4, Thermo Scientific, 2001) for the FIDs and ECDs signal and with Agilent Chemstation (MSD Chemstation, D.02.00.275, Agilent Technologies 1989–2005) for the MS signal. Each chromatographic peak was individually inspected, and the baseline was adjusted when the integrated area was not accurately integrated by the software. Concentration values (pptv) were converted to mass concentrations (μg VOC puff^–1^) (SI Appendix B). Details on the standards and procedures for the VOC calibration are described in Simpson et al. (2020).^49^ Briefly, the detector response in area units is converted into mixing ratios by using a response factor calculated from a system of multiple standards, including calibrated standards and working standards. Unlike in the PTR-ToF-MS, baseline breath values were not subtracted from exhaled breath values, as two separate WAS canisters were used for the sampling, and signals from the different breath types were not additive in this case.

To compare concentration responses between the PTR-ToF-MS and the GC platform, a multicomponent calibration mix was run on both instruments and compared with certified concentrations. The ambient air quality gas standard (Cylinder CC302254, 2000 psi, AiR Environmental Inc.) was composed of acetaldehyde, methanol, ethanol, acrolein, propanal, acetone, 2-propanol, acetonitrile, methyl *tert*-butyl ether, methacrolein, methyl vinyl ketone, methyl ethyl ketone, and 1-butanol. Table S3 presents the concentrations measured by GC and PTR-ToF-MS instruments and the concentrations certified by the manufacturer.

### Propylene Glycol and Glycerol Standard Experiments

To assess the thermal degradation of the e-liquid components, pure PG (Fisher, > 99%, CAS 57-55-6) and GLY (Fisher, > 99.9%, CAS 56-81-5) decomposition products were sampled by both the PTR-ToF-MS and the WAS canisters. Two 1 L sampling bags were made from food-grade nylon-coated polyethylene film (FoodSaver) and used for each humectant experiment. Participant 5′s *open* vape device (SMOK Morph 2) was used for these experiments. The liquid cartridge was cleaned with isopropanol, ethanol, and nanopure water and then dried thoroughly prior to the humectant being added. New cotton and coils were used for each humectant experiment. As both PG and GLY are highly viscous (55 and 1470 mPa·s, respectively, at 293 K),^50,51^ we allowed ample time for the interior cotton wick to absorb humectant prior to turning the atomizer on. The vape was set to 20 W, 0.43 Ω, and 2.92 V, the same settings used by participant 5. Prior to each experiment, 800 mL of clean air was added to the bag. Once the pure humectant was loaded into the vape device, the atomizer was turned on and the heated PG (or GLY) aerosol was collected using a clean disposable syringe. The bag was prefilled with clean air, and 5 mL of heated humectant aerosol was injected into the bag that was sampled with both the PTR-ToF-MS and WAS canisters for respective analysis.

## Results and Discussion

### Overall Comparison between Exhaled Puff and Direct Vape Aerosol

**Figure 2:**
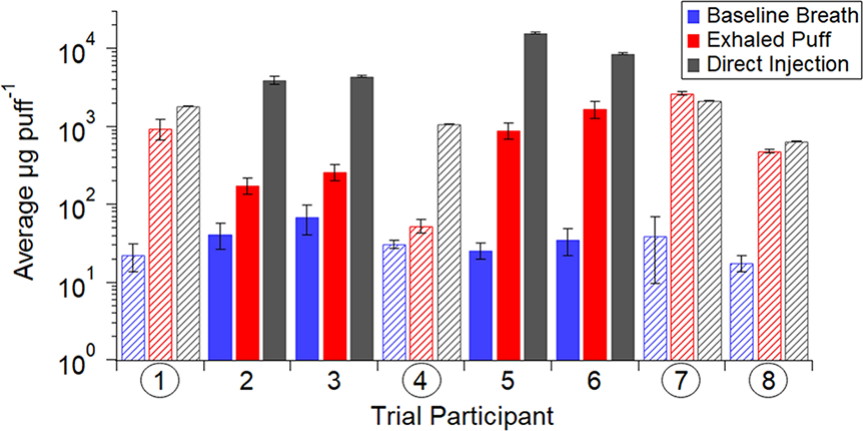
Combined mass concentrations of the species detectable by PTR-ToF-MS for all participant visits. The average mass per puff (μg VOC puff^–1^) is shown for baseline breath (no vape, blue), exhaled puff (after inhaling from the vape device, red), and direct injection of vape aerosol (gray). The variability in the emissions is represented by error bars (one standard deviation) calculated using each participant’s puff topography data (Table S1). Note that the vertical scale is logarithmic. Participants 1, 4, 7, and 8 used *closed* vapes (circled numbers, striped bars), whereas participants 2, 3, 5, and 6 used *open* vapes (solid bars). A linear version of this figure is presented in the SI (Figure S3).

Figure 2 shows the combined mass concentrations of the species observed by the PTR-ToF-MS for all participant visits. These values were calculated by summing the mass concentrations for all detected compounds (SI Table S4) and then averaging across all five visits per participant and breath type (baseline breath, exhaled puff, and direct injection). For all participants, baseline breath measurements were low, as expected by sampling protocols prohibiting eating, drinking, or smoking prior to the visit. Averaged mass concentrations for the exhaled puff were lower than direct injection measurements, indicating significant VOC absorption by participants prior to exhalation. This was the case for all participants except for participant 7. It is likely that this participant’s exhaled volumes were inconsistent and resulted in uncertainty in corrected direct injection values calculated with eqs S5 and S6.

For participants who smoked *open* vapes (participants 2, 3, 5, and 6), VOC mass concentrations in the direct injection measurements (Figure 2, gray solid bars) were an order of magnitude higher than exhaled puffs (Figure 2, red solid bars). Those who vaped *open* devices likely operated them at higher power outputs than participants who vaped with *closed* devices (Table S1) resulting in a more concentrated puff. In addition, these individuals inhaled larger volumes and therefore, produced a higher concentration of VOCs in the bag. Participant 5 is a noticeable example of this, as the *open* vape device was set to 140 W and the participant inhaled an average of 260 ± 40 mL with high VOC concentrations (Participant 5 direct injection, Figure 2). High VOC concentrations at high vape wattage are consistent with Gillman et al. (2016) who showed that increases in power applied to vape device coils correlated with increases in the total VOC yield.^52^ In general, participants who smoked *closed* vapes (1, 4, 7, and 8) inhaled lower puff volumes (Table S1 and Figures 2 and S3) that were less concentrated, as shown by lower mass concentrations in direct injection measurements. Figure S4 further presents this puff volume and mass concentration distinction among *closed* (red markers) and *open* (blue markers) vape devices measured by the PTR-ToF-MS. For all 12 selected VOCs presented in Figure S4, *closed* vape devices have smaller puff volumes with mass concentrations less widespread than *open* vape devices. In summary, the participants using *open* vape devices inhaled and absorbed a higher dose of total VOCs compared to the *closed* vape participants.

Figure S5 summarizes results for twenty-six selected VOCs averaged across all participant collections for baseline breath, exhaled puff, and direct injection measured by the PTR-ToF-MS. All presented VOCs follow the same trend of exhaled puff mass concentrations being lower than direct injection values due to the efficient deposition of VOCs in the respiratory tract. Thermal degradation of humectants PG and GLY are known to produce carbonyl products including formaldehyde, acetaldehyde, acetone, and acrolein.^53,54^ Acetaldehyde and acetone/propanal had the largest apparent concentrations across all participant visits in all breath types (Figures S4a,b and S5a). A few compounds are slightly elevated in baseline breath. For example, acetone is known to be a byproduct of fat metabolism processes in the liver (typically 1 ppmv),^55^ and acetone concentrations are known to increase during exercise.^56^ Participants in this study walked approximately 1000 ft from the parking lot to the laboratory prior to sampling and hence this could have impacted baseline acetone levels. Other slightly elevated VOCs present in the baseline breath are also major endogenous breath metabolites.^57−59^

All e-liquids utilized in this study contained various concentrations of nicotine (Table S1). Nicotine (*m*/*z* 163.1230, C_10_H_15_N_2_^+^) was present in the exhaled breath of only nine trials (one trial of participant 2, all trials of participant 3, and three trials of participant 7). Nicotine was not detected in the baseline breath (Figure S5b), but for trials where nicotine was observed, the direct injection concentrations were an order of magnitude higher than the exhaled breath concentrations. On average, ∼10 μg puff^–1^ of nicotine was measured in the exhaled breath, whereas ∼100 μg puff^–1^ was detected in the direct injection measurements (mimicking inhalation), indicating high nicotine absorptivity by participants prior to exhalation.^46,60^ This result is widely supported by previous studies that measured enhanced plasma nicotine concentrations in human participants after vaping, with over 99% of nicotine being retained after inhalation.^37,61−63^

### Effect of Vape Flavor on Exhaled Breath VOC Distribution Measured by PTR-ToF-MS

PTR-ToF-MS mass spectra for all participants’ exhaled breaths (Figures S6–S13) along with a list of observed ions (Table S4) can be found in the SI. Potential assignments for ions observed in this study are consistent with previously reported assignments from PTR-ToF-MS measurements.^40,45,46,60,64−78^ Across all 40 exhaled breath mass spectra, there was a minimal deviation in mass spectral peaks and ten ions reproducibly dominated the signal. Ions at nominal *m*/*z* 31, 41, 43, 45, 47, 57, 59, 61, 75, and 93 were the major ions commonly observed. An exception to this is participant 2’s mass spectra for mint and tobacco-flavored trials (Figure S7a,b). These trials had low signal and the dominant peaks were acetone (*m*/*z* 59) and isoprene (*m*/*z* 69), normal baseline substituents of human breath.^79−81^ Participants 2 and 3 smoked the same vape device and e-liquids, but in comparison to participant 3, participant 2 generated lower intensity mass spectra (Figure S7) for mint and tobacco trials, and mass spectral peaks did not resemble other participant trials. This result reflects the variability in emissions depending on how a participant interacts with their vape device and how personal habits influence VOC mass concentrations.^82^

**Figure 3:**
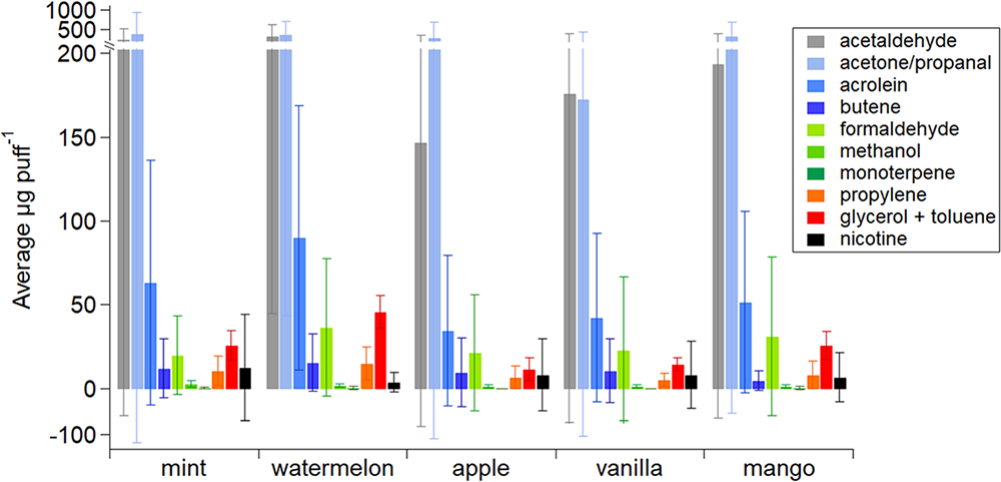
PTR-ToF-MS results of selected VOCs from participant exhaled puffs as a function of e-liquid flavor class. These results were averaged among all 40 participant trials. Error was calculated using each participant’s puff topography data which varied by visit (Table S1).

Five different vape flavor profile groups (mint, watermelon (or tobacco), apple, vanilla, and mango) were selected for this study based on population prevalence. Figure 3 shows the PTR-ToF-MS average mass concentrations of the ten most abundant VOCs from all participants’ exhaled puffs as a function of the e-liquid flavor class. All flavor profiles exhibited the same trend: acetaldehyde (gray), acetone/propanal (lightest blue), and acrolein (medium blue) were the largest peaks. Blair et al. (2015) utilized the same PTR-ToF-MS instrument for VOC analysis of an e-cigarette and compared results to traditional cigarettes.^36^ That study did not utilize participants or multiple vape devices but reported values were comparable to those in Figure 3 for acetaldehyde (∼100 μg VOC cig^–1^) and acrolein (∼30 μg VOC cig^–1^).^36^ As the present study surveyed far more variables (participant interactions with vape devices, 40 different vape devices, five different flavor profiles, etc.), our reported values and errors are larger than those in Blair et al. (2015).

Flavor compounds, which are often esters, were not observed to be the main contributors to signal in PTR-ToF-MS measurements. Buhr et al. (2002) tested the PTR-MS fragmentation patterns of 17 esters and found that the molecular [M + H]^+^ ion itself and/or the corresponding protonated acids ions (e.g., for all acetate esters, the acetic acid parent ion at *m*/*z* 61, and its corresponding fragment at *m*/*z* 43) dominate the mass spectra, with the protonated acid often being the main fragment.^72^ Fragmentation of acetate esters is supported by our results with acetic acid mass concentrations detected up to ∼35 μg puff^–1^ (*m*/*z* 61.0284) in the exhaled breath, with its fragment (*m*/*z* 43.0178) detected up to ∼14 μg puff^–1^ (Figure S5c). Note that fragmentation of glycolaldehyde, a thermal degradation product of GLY, does not contribute significantly to these ions, as described below. We did not observe ester [M + H]^+^ ions as main contributors to participant mass spectra, with the exception of a small amount of methyl butanoate at *m*/*z* 103.0754 (C_5_H_11_O_2_^+^) and propyl butanoate at *m*/*z* 130.1067 (C_7_H_15_O_2_^+^). In addition, previous studies have shown toxic aldehyde flavorants, such as cinnamaldehyde, diacetyl, acetoin, maltol, and benzaldehyde, were present in e-cigarette liquids.^83−85^ After e-liquid thermal degradation, it has been shown that small aldehydes are produced during the thermal decomposition of flavorants.^86−88^ Exhaled breath results shown in Figure 3 support previous work reporting the detection of acetaldehyde, acetone/propanal, acrolein, and formaldehyde after the heating of flavored e-liquid followed by subsequent inhalation and exhalation.

### Fragmentation of Humectants in PTR-ToF-MS Measurements

**Figure 4:**
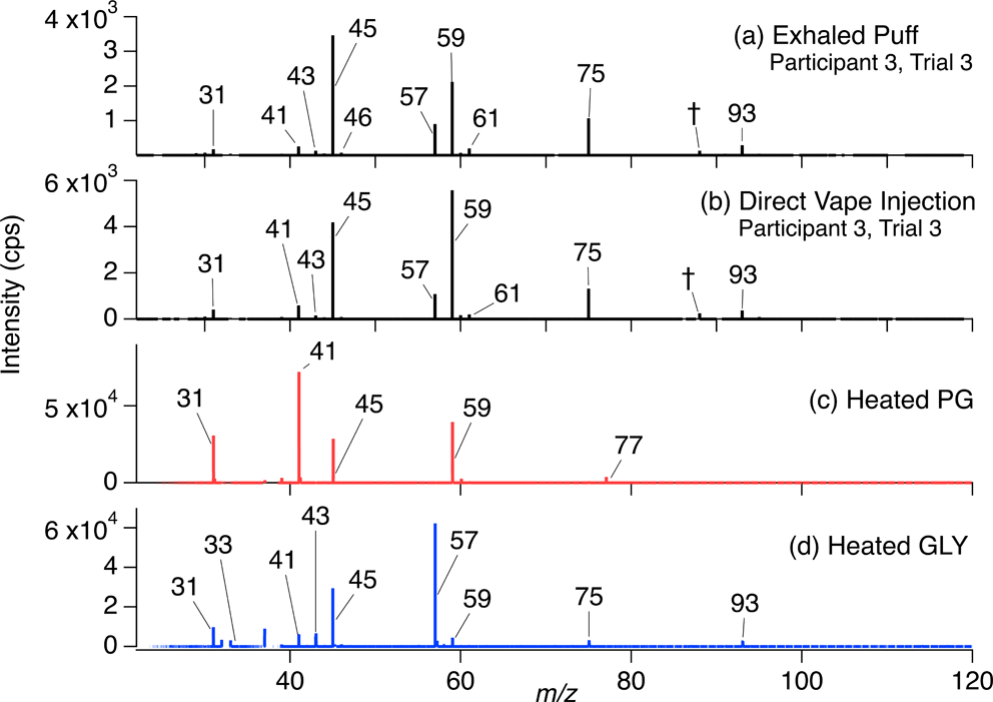
Typical unit mass resolution PTR-ToF-MS mass spectra from (a) exhaled vape puff of participant 3 in trial 3, (b) direct vape injection from vape smoked by participant 3 in trial 3, (c) heated propylene glycol (PG) (Fisher, > 99%, CAS 57-55-6), and (d) heated glycerol (GLY) (Fisher, > 99%, CAS 56-81-5). Both humectants were aerosolized separately using participant 5′s *open* vape (SMOK Alike). The peak indicated with (†) corresponds to DMAC at *m*/*z* 88, a known impurity of Tedlar bags. The identity of labeled ions can be found in Table 1.

To better understand the potential role of humectants in our observations, we conducted control experiments with PG and GLY standards. Figure 4 shows the mass spectra of participant 3′s third trial exhaled puff (a) and direct injection (b) compared to heated pure PG (c) and GLY (d). A compilation of both heated humectant spectra comprises the exhaled puff and direct injection spectra for participant 3, trial 3, and all other trials (Figures S6–S13). Simultaneously, two different processes can be the source of the ion distribution observed in the PTR-ToF-MS mass spectra: (1) fragmentation of humectant molecules within the PTR-ToF-MS ion source, and (2) thermal decomposition of the humectant within the vape device. Indeed, during proton transfer reaction ionization, alcohols are known to be protonated by H_3_O^+^ then undergo loss of a water molecule and leave behind a hydrocarbon ion.^89^ During these fragmentation reactions, only a small fraction of the parent ion is detectable. On the other hand, thermal degradation of PG and GLY is known to occur by two pathways: heat-induced dehydration and H-abstraction by radicals (OH) (followed by oxidation and bond cleavages) that can generate different products depending on the parent molecule.^90,91^ Products formed through the free radical reaction pathways are enhanced with increased combustion temperature, as this generates more free radicals.^92^

In the case of PG (CH_3_CH_2_(OH)CH_2_OH, MW = 76 g mol^–1^), we observed ions at nominal masses *m*/*z* 31, 41 (largest), 45, 59 (second largest) and 77 (Figure 4c and Table 1). PG’s protonated [M + H]^+^ ion (*m*/*z* 77) is known to fragment (during ionization) with 95% efficiency into *m*/*z* 59.0491 (C_3_H_7_O^+^).^46,76,89^ This is consistent with our observation of a small *m*/*z* 77.0597 parent ion peak and a much larger *m*/*z* 59.0491 peak, as shown in Figure 4c. Additionally, the heat-induced dehydration reaction of PG is known to produce acetone and propanal, which are structural isomers and would both be detected by the PTR-ToF-MS at *m*/*z* 59.0491.^13,93,94^ Another thermal degradation product, corresponding to 1,2-propadiene (e.g., loss of two water molecules), is observed at *m*/*z* 41.0386 (C_3_H_5_^+^) as the largest ion in the PG heated spectrum (Figure 4c). Formaldehyde (*m*/*z* 31.0178) and acetaldehyde (*m*/*z* 45.0335) are known products of the C–C bond cleavage during the OH radical reaction with PG.^93^

**Table 1:** Prominent Ions Observed in the PTR-ToF-MS Mass Spectra (Figure 4) from Heated Humectant Control Experiments of Propylene Glycol (PG) and Glycerol (GLY)^a^

*m*/*z*	empirical formula	potential assignment	heated PG	heated GLY
31.0178	CH_3_O^+^	formaldehyde	X	X
33.0335	CH_5_O^+^	methanol	X (small)	X
41.0386	C_3_H_5_^+^	PG – 2(H_2_O); 1,2-propadiene	X	X
43.0178	C_2_H_3_O^+^	glycolaldehyde fragment	X (small)	X
43.0542	C_3_H_7_^+^	propene	X (small)	X
45.0335	C_2_H_5_O^+^	acetaldehyde	X	X
57.0335	C_3_H_5_O^+^	GLY – 2(H_2_O); acrolein		X
59.0491	C_3_H_7_O^+^	PG – H_2_O; acetone/propanal	X	X
61.0284	C_2_H_5_O_2_^+^	glycolaldehyde		X (small)
75.0441	C_3_H_7_O_2_^+^	GLY – H_2_O; hydroxyacetone		X
77.0597	C_3_H_9_O_2_^+^	PG	X	
93.0546	C_3_H_9_O_3_^+^	GLY		X

a Structural isomers cannot be distinguished using the PTR-ToF-MS; hence, multiple compound assignments are given for certain peaks.

In the case of GLY (CH_2_(OH)CH_2_(OH)CH_2_OH, MW = 92 g mol^–1^), we observed ions at nominal masses *m*/*z* 31, 33, 41, 43, 45 (second largest), 57 (largest), 59, 75, and 93 (Figure 4d and Table 1). As with PG, we see a small GLY protonated parent ion peak at *m*/*z* 93.0546 (C_3_H_9_O_3_^+^). The largest peak in this spectrum, at *m*/*z* 57.0335, is attributed to the parent ion of acrolein (C_3_H_4_O), the GLY thermal degradation product with the loss of two water molecules.^90,93^ The small peak at *m*/*z* 75.0441 can be attributed to the parent molecular ion of the first thermal decomposition product, hydroxyacetone (C_3_H_6_O_2_), which is a less preferred product of GLY heating.^93^ Additional thermal decomposition products, formaldehyde and acetaldehyde, are known to form through the GLY C–C bond cleavage after the loss of water and are observed in our mass spectrum at *m*/*z* 31.0178 (CH_3_O^+^) and 45.0335 (C_2_H_5_O^+^), respectively.^90,93,94^ Proposed to be formed from the thermal degradation of GLY,^94^ glycolaldehyde (C_2_H_4_O_2_) is observed (with low intensity) at *m*/*z* 61.0284 (C_2_H_5_O_2_^+^) and its fragment ion at *m*/*z* 43.0178 (C_2_H_3_O^+^). Additionally, gas phase fragmentation of the metastable protonated glycerol ion was previously reported to result in major product ions at nominal masses *m*/*z* 75 (C_3_H_7_O_2_^+^), 57 (C_3_H_4_^+^), 61 (C_2_H_5_O_2_^+^), 45 (C_2_H_5_O^+^) and 31 (CH_3_O^+^).^95^ All of these ions were observed in our mass spectra and could originate from the fragmentation of GLY upon ionization inside the ion source, adding to the complexity of the MS spectra. Methanol (*m*/*z* 33), 1,2-propadiene (*m*/*z* 41), and acetone/propanal (*m*/*z* 59) were also observed, but it is unknown whether these are GLY thermal decomposition products or produced within the PTR-ToF-MS ionization source.

### Comparison of the PTR-ToF-MS Results with Off-Line GC Measurements

As the PTR-ToF-MS results are overwhelmed by humectant fragmentation and thermal decomposition products, we sought to compare our results to those measured by GC, which are not subject to ion fragmentation (but could potentially be affected by wall losses of compounds in the WAS canisters). Figure S14 shows the relationship between reported PTR/GC exhaled breath concentrations for 12 VOCs detected by both instruments. The gray diagonal lines represent an ideal 1:1 relationship between measured concentrations. For all 12 compounds, the best-fit lines between the PTR-ToF-MS and GC (black, dashed lines) deviate substantially from the 1:1 line and the data are overall poorly correlated. Except for isoprene, monoterpene, and ethanol, PTR-ToF-MS reports much higher values than GC (Figure S14). Ethanol exhibits the strongest correlation between measurements, however, the PTR *underestimates* the GC values by a factor of 10. Note that ethanol was successfully identified and quantified in all WAS samples using GC-FID, as coeluting acetonitrile was not detected. This result was confirmed by GC-MS analysis. The comparison with a certified cylinder containing a VOC mixture showed that when using exclusively *m*/*z* 47 for quantification, the ethanol signal was underestimated by the PTR-ToF-MS by approximately the same factor (Table S3). Inomata et al. (2009) showed that the signal at *m*/*z* 47 for ethanol decreases as the ratio of the drift tube electric field strength (*E*) to the buffer gas number density (*N*) value increases.^96^ For *E/N* values similar to the ones used in this study (∼132 Td), the fragmentation of the parent ion is thought to produce H_3_O^+^ ions which are impossible to quantify as they correspond to the reagent ions. This leads to a large underestimation of ethanol overall by the PTR-ToF-MS.

Monoterpenes and isoprene fragmentation have been previously reported to occur in the PTR-ToF-MS.^97,98^ Accounting for fragments for all of these species would certainly help to close the gap between the two types of measurements; however, assigning fragments in a complex mixture such as vape aerosol is challenging. PTR-ToF-MS values of toluene were up to two orders of magnitude higher compared to that reported by the GC, further enforcing quantitative measurement of toluene in the PTR-ToF-MS is impacted by GLY at the same nominal mass (Figure S14) in any vape aerosol samples. Compounds associated with humectant fragmentation in PTR-ToF-MS (acetaldehyde, acetone, methanol) had poor correlation with GC measurements, and the PTR-ToF-MS values are systematically 3 orders of magnitude larger than reported by GC. While the calibration plots in Figure S1 demonstrate that the PTR-ToF-MS can measure these compounds reasonably well, the dramatic deviation between the PTR-ToF-MS and GC data reflects the strong impact of fragmentation within the PTR ionization source.

Potentially, other sampling artifacts may have contributed to the discrepancies observed. These include the potential revolatilization of smaller VOCs (from particles) when entering the slightly heated PTR-ToF-MS inlet (70 °C) from the room-temperature bag. The sample residence time in this section is relatively short (∼1 s) and the temperature is relatively low that revolatilization is not expected to be a dominant factor. On the other end, the exhaled aerosol was humid, and it is possible that there were additional wall losses inside the WAS canisters, in addition to potential partitioning of the VOCs onto the particles collected on the wall of the canisters. Note that the canisters remained at room temperature from sampling to analysis, which may favor partitioning. The canisters were analyzed right after the sampling to mitigate any adsorption artifacts. Although these artifacts may contribute some to the discrepancies observed between the two techniques, they do not solely explain the order of magnitude difference observed for most VOCs, and the fragmentation of the humectant is largely responsible for this difference.

**Figure 5:**
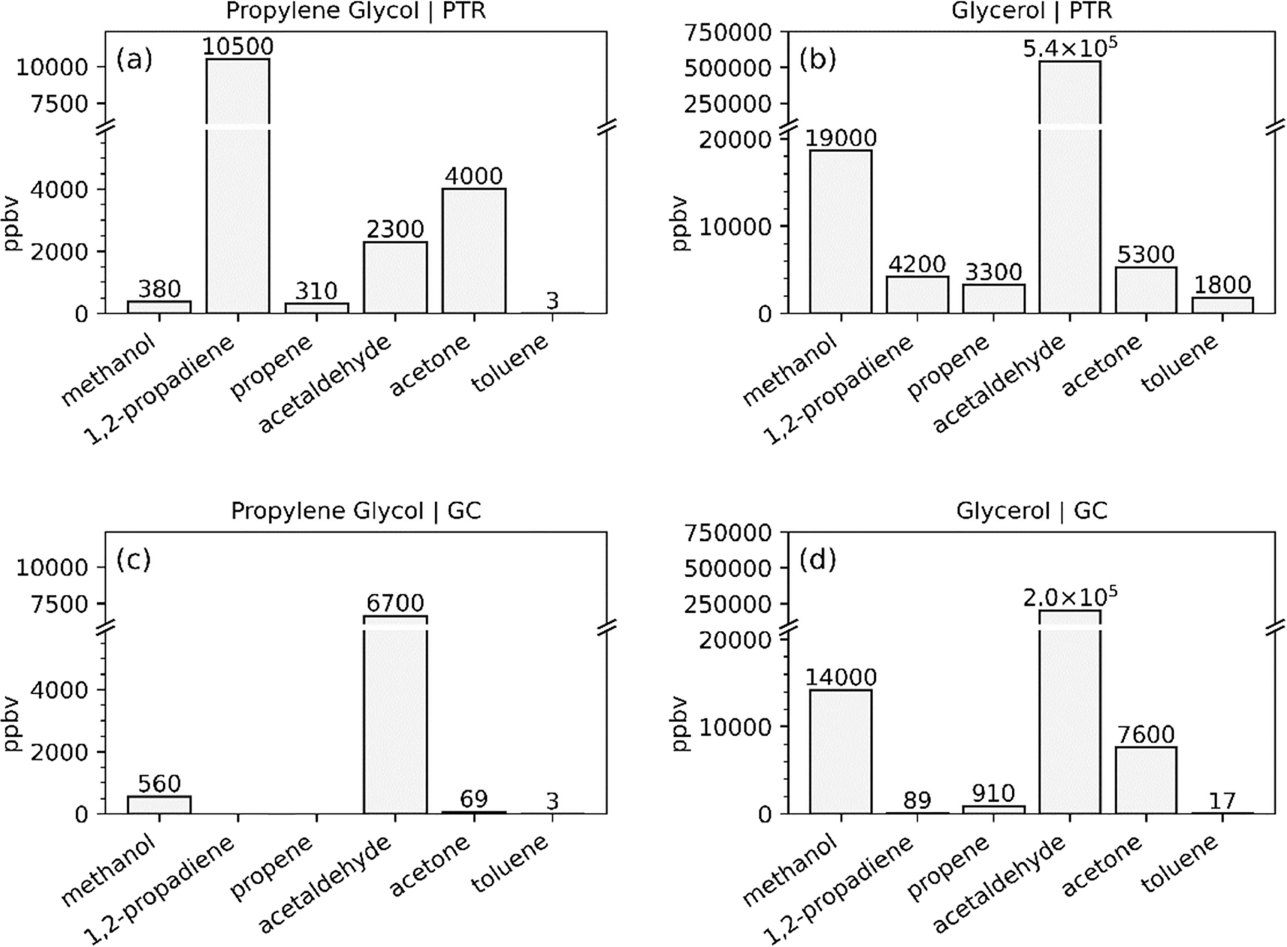
Reported mixing ratios for methanol, 1,2-propadiene, propene, acetaldehyde, acetone, and toluene by PTR-ToF-MS (a, b) and GC (c, d) from the control experiments with heated pure solvents. In panels (a) and (b) “acetone” refers to *m*/*z* 59, which is the sum of acetone and propanal; “toluene” refers to *m*/*z* 93, which is the sum of glycerol and toluene; and “1,2-propadiene” refers to the sum of 1,2-propylene and the C_3_H_5_^+^ fragment ion from PG ([M+H-2H_2_O]^+^).

Figure 5 shows the reported mixing ratios (in ppbv) for compounds detected by both PTR-ToF-MS and GC for the control experiments with heated PG and GLY. Methanol (*m*/*z* 33) and acetaldehyde (*m*/*z* 45) are of the same order of magnitude between instruments and solvent cases, indicating that these compounds are solely attributed to thermal decomposition. 1,2-Propadiene (*m*/*z* 41) and propene (*m*/*z* 43) were not detected by GC for the PG experiment (Figure 5c), further supporting fragmentation within the PTR-ToF-MS ionization source leading to orders of magnitude enhancement of these compounds in our participant data set. For PG, acetone is 2 orders of magnitude larger in the PTR-ToF-MS (Figure 5a) compared to GC measurements (Figure 5c). Values are of the same order of magnitude in the GLY experiment, confirming that acetone is a thermal decomposition product from both humectants, but it is also a fragmentation product from PG, further increasing the acetone response in exhaled breath measurements in the PTR-ToF-MS. Toluene is measured by both instruments at 3 ppbv for PG. In the GLY experiment, PTR-ToF-MS reports toluene at 1800 ppbv whereas GC reports 17 ppbv. This discrepancy is attributed to the GLY parent ion overlapping with toluene at the same nominal mass *m*/*z* 93 (Figure S2). Since PG does not have a peak overlap at this mass, there was no interference in the measured toluene concentration by the PTR-ToF-MS.

## Conclusions

Real-time measurements of inhaled and exhaled VOCs offer an attractive way to assess the exposure of bystanders to second-hand vape smoke. However, we found that PTR-ToF-MS alone, a state-of-the-art online VOC detector, may not be suitable for this task due to complex patterns of thermal decomposition of humectants in the vaping devices and ion fragmentation in the PTR-ToF-MS ion source. We observed large differences, sometimes by as much as 2 orders of magnitude, between VOC concentrations measured by offline GC and online PTR-ToF-MS instruments. As the e-liquids used in this study were a mixture of PG and GLY, a combination of effects explored in the pure humectant experiments (Figure 5) contributed to this large discrepancy in the human trial data set. Together, the fragmentation of humectant in the PTR-ToF-MS ion source, the generation of pyrolysis products during vaping, and the lack of accurate ingredient labeling of e-liquids has made it challenging to interpret observations regarding flavorings and other potentially toxic species associated with secondhand vape exposure.

Comparison of PTR-ToF-MS measurements with those of a GC platform (GC-MS, GC-FID, GC-ECD) indicates that *quantitative* results should be cautiously interpreted. Ionization within the PTR-ToF-MS leads to overestimation of a range of VOCs, including some like acrolein and acetaldehyde which have potentially significant health implications and would result in misrepresentation of the potential for vape emissions on adverse health effects. Values reported herein from PTR-ToF-MS measurements should be used with caution by experts in the field to influence public health policy on the dangers of vaping, as these values are inflated for analytical reasons described in the discussion section. The WAS canisters coupled with a GC analysis, though it is an offline technique and requires more time to run than the PTR-ToF-MS, provide more accurate quantification of VOCs in vape-related emissions. Lower VOC values reported by GC, as shown in Figure S15, are more representative of what an individual is exhaling during a vaping event. Furthermore, *quantitative* e-cigarette results reported by PTR-ToF-MS measurements of Blair et al. (2015) are likely overestimated and should be considered carefully.^36^ If future studies wish to use PTR-ToF-MS for *quantitative* purposes in vaping trials, analysis of e-liquid components (prior to combustion) and control studies using PTR-ToF-MS (accounting for fragmentation effects) could provide a template for *a quantitative* framework that could then be applied to participants smoking the same device/e-liquid.

While one must be careful when drawing *quantitative* conclusions from PTR-ToF-MS measurements, PTR-ToF-MS can provide valuable *qualitative* results in a human trial vaping study. Overall, this study indicates that there is substantial retention of organic species in the lungs of individuals, which selectively changes the composition of exhaled breath compared to the inhaled vape aerosol. However, VOCs in the exhaled breaths are still enhanced relative to the baseline breaths, illustrating the need to quantify the burden that vaping has on secondhand exposure.

This study was purposely designed without strict participant constraints to best survey realistic secondhand vape exposure. To better constrain fragmentation issues in PTR-ToF-MS measurements, more controlled and reproducible samples would be required, however, these would not reflect the diversity of vaping behavior. Nevertheless, PTR-ToF-MS fragmentation effects elucidated in this study are independent of participant variables and should be considered in future works.

## Supporting Information Available

Information on participants’ puff topography and vaping devices, additional details about PTR-ToF-MS and WAS measurements, amounts of exhaled VOCs measured by PTR-ToF-MS and GC, list of detected ions, PTR-ToF-MS mass spectra for all participants and visits, and comparison of exhaled amounts measured by PTR-ToF-MS and WAS (PDF)
