# Supplementary material for: Chemical Analysis of Exhaled Vape Emissions: Unraveling the Complexities of Humectant Fragmentation in a Human Trial Study
Source: Chem Res Toxicol. 2024 May 21;37(6):1000–10. doi: 10.1021/acs.chemrestox.4c00088 (PMC11187636; doi:10.1021/acs.chemrestox.4c00088)
Supplement: Supplementary file 1 — tx4c00088_si_001.pdf [file tx4c00088_si_001.pdf]

Supporting Information

Chemical Analysis of Exhaled Vape Emissions: Unraveling the Complexities of Humectant Fragmentation in a Human Trial Study

Katherine S. Hopstock,[1] Véronique Perraud,[1] Avery B. Dalton,[1] Barbara Barletta,[1] Simone Meinardi,[1] Robert M. Weltman,[2] Megan A. Mirkhanian,[2] Krisztina J. Rakosi,[1] Donald R. Blake,[1] Rufus D. Edwards,[2] and Sergey A. Nizkorodov[1]

[1] Department of Chemistry, University of California, Irvine, Irvine, CA 92697, USA.
[2] Program in Public Health, University of California, Irvine, Irvine, CA 92697, USA.

* Corresponding authors' e-mails: edwardsr@hs.uci.edu and nizkorod@uci.edu

This document contains the following: Information on participants' puff topography and vaping devices, additional details about PTR-ToF-MS and WAS measurements, amounts of exhaled VOCs measured by PTR-ToF-MS and GC, a list of detected ions, PTR-ToF-MS mass spectra for all participants and visits, a comparison of exhaled amounts measured by PTR-ToF-MS and WAS.

Table of Contents

Table S1. Participants, vape devices, e-liquid, and inhaled puff information. ... 3

Appendix A. ... 6

Figure S1. PTR-ToF-MS calibration ... 7

Figure S2. Expanded view of the PTR-ToF-MS spectra ... 11

Figure S3. Liner scale of the combined mass concentrations ... 12

Appendix B. Whole Air Sampling Canisters and Gas Chromatography Systems Information ... 13

Table S2. VOC limit of detection and measurement precision and accuracy for the GC platform. ... 14

Table S3. Measured concentrations of a 13-component ambient air quality gas standard ... 15

Figure S4. Exhaled puff PTR-ToF-MS values ... 16

Figure S5. PTR-ToF-MS results of selected VOCs ... 17

Table S4. Ions observed in the PTR-ToF-MS mass spectra ... 18

Figure S6. PTR-ToF-MS mass spectra for Participant 1's five visits ... 20

Figure S7. PTR-ToF-MS mass spectra for Participant 2's five visits ... 21

Figure S8. PTR-ToF-MS mass spectra for Participant 3's five visits ... 22

Figure S9. PTR-ToF-MS mass spectra for Participant 4's five visits ... 23

Figure S10. PTR-ToF-MS mass spectra for Participant 5's five visits ... 24

Figure S11. PTR-ToF-MS mass spectra for Participant 6's five visits ... 25

Figure S12. PTR-ToF-MS mass spectra for Participant 7's five visits ... 26

Figure S13. PTR-ToF-MS mass spectra for Participant 8's five visits ... 27

Figure S14. Scattered data for each PTR/GC exhaled breath measurement ... 28

Figure S15. Exhaled puff GC values ... 29

References ... 30

Table S1. Participants, vape devices, e-liquid, and inhaled puff information. Vapes are classified as either *closed* or *open*. Participant preferences and operating inputs (power, resistance, and voltage settings) are reported for *open* vapes. Flavors denoted with (*) were conducted instead of watermelon. Flavors denoted with (**) are alternatives for vanilla. The reported inhaled puff volumes are reported as the average amongst all puffs during one visit with uncertainty values reported to one standard deviation. Stated nicotine concentrations are those reported by the manufacturer, whereas actual nicotine concentrations are those determined by GC-MS measurements. E-liquid nicotine measurements were conducted using the method described in Pagano *et al.* (2015) for thirty three of the forty e-liquids included in this study.[1] Gas chromatograph (6850 Series II, Network GC System, Agilent Technologies) coupled to a quadrupole mass spectrometer in electron ionization mode (5975B VL MSD, Agilent Technologies) was used to analyze the nicotine standards and e-liquid samples. Nicotine values typically deviated from those reported by the manufacturers.

Participant & Vape Type	E-Cigarette Brand/Model	E-Liquid Flavor	Inhaled puff volume (mL)	Stated nicotine (mg/mL)	Actual nicotine (mg/mL)	Set Power (Watt)	Resistance (Ω)	Voltage (V)
1 *Closed*	PUFF Xtra Limited	Cool Mint	55 ± 7	50	–	N/A	N/A	N/A
	PUFF Xtra Limited	Watermelon Skittles	36 ± 2	50	–			
	Puffs Plus	Mango	61 ± 9	50	33			
	Puffs Plus	Banana Vanilla	70 ± 10	50	–			
	Puffs Plus	Apple	59 ± 10	50	23			
2 *Open*	SMOK® POZZ X	Spearmint (*Mints*)	201 ± 160	50	61	40	0.4	4
		Apple (*Red's Apple by 7 Daze Salt Series*)	406 ± 150	50	58	5	0.4	1
		Sweet and Salty Tobacco*	138 ± 50	50	–	9	0.66	2.44
		Vanilla Custard (*BLVK® Nicotine Salt*)	331 ± 30	50	85	6	0.39	1.53
		Mango (*Skwezed Salt Based Nicotine*)	297 ± 120	50	79	6	0.38	1.52

3 *Open*	SMOK® POZZ X	Spearmint (*Mints*)	142 ± 10	50	61	40	0.4	4
		Apple (*Red's Apple by 7 Daze Salt Series*)	262 ± 40	50	58	5	0.4	1
		Sweet and Salty Tobacco*	204 ± 10	50	–	9	0.66	2.44
		Vanilla Custard (*BLVK® Nicotine Salt*)	233 ± 20	50	85	6	0.39	1.53
		Mango (*Skwezed Salt Based Nicotine*)	262 ± 10	50	79	6	0.38	1.52
4 *Closed*	LUCID AIR	Watermelon Ice	86 ± 10	50	41	N/A	N/A	N/A
	LUCID AIR	Jazzy Mango	65 ± 3	50	43			
	LUCID AIR	Apple Ice	38 ± 2	50	53			
	LUCID AIR	Icy Mint	64 ± 5	50	–			
	Blu	Vanilla	31 ± 8	25	27			
5 *Open*	SMOK® Morph 2	Apple (*Red's Apple by 7 Daze*)	254 ± 30	6	4	140	0.36	7.09
		Vanilla custard (*Vapetasia*)	341 ± 70	6	4	140	0.36	7.09
		Mango (*Red's Apple by 7 Daze*)	296 ± 9	6	5	140	0.36	7.09
		Jewel Mint (*Pod Juice*)	203 ± 20	6	2	140	0.36	7.09
		Watermelon (*Red's Apple by 7 Daze*)	283 ± 50	6	5	140	0.36	7.09
6 *Open*	SMOK® Alike	Watermelon (*Red's Apple by 7 Daze*)	179 ± 30	3	2	24	0.43	3.19
		Mango (*Red's Apple by 7 Daze*)	196 ± 30	3	2	20	0.43	2.92
		Apple (*Red's Apple by 7 Daze*)	262 ± 20	3	3	15	0.43	2.52
		Vanilla (*Custard Monster*)	259 ± 30	3	3	15	0.43	2.52
		Mint Ice (*Red's Apple by 7 Daze*)	243 ± 10	3	3	15	0.43	2.52
7 *Closed*	Elfbar BC5000	Watermelon Bubble Gum	127 ± 30	50	59	N/A	N/A	N/A
	Elfbar BC5000	Mango Peach	129 ± 10	50	61			
	Hyde Rebel Pro	Vanilla	153 ± 10	50	–			
	Elfbar BC5000	Lemon Mint	114 ± 10	50	67			
	Elfbar BC5000	Sour Apple	122 ± 20	50	61			

8 *Closed*	Flum Gio	Juicy Apple	36 ± 4	50	95	N/A	N/A	N/A
	Flum Gio	Tobacco Cream**	44 ± 9	50	99			
	Flum Float	Watermelon Lush Ice	54 ± 10	50	74			
	Flum Float	Cool Mint	53 ± 6	50	73			
	Flum Float	Strawberry Mango	44 ± 3	50	103			

10

Appendix A. Proton-transfer-reaction time-of-flight mass spectrometer (PTR-ToF-MS) information.

Appendix A.1. General Description of Ionization in PTR-ToF-MS

PTR-ToF-MS is a soft chemical ionization instrument. Ionization occurs through proton transfer reactions with volatile organic compounds (VOCs) and reagent hydronium ions (H_3O^+) to form $[M+H]^+$ ions. A hallow-cathode plasma ion source produces H_3O^+ reagent ions that enter a reaction chamber (drift tube) for exposure and collision with analyte VOC molecules. Proton transfer reaction, and subsequent ion detection by the mass spectrometer, will occur during collision only if the proton affinity (PA) of the analyte compound is greater than that of water (691 kJ mol^{-1}).[2] Common constituents of air (N_2, O_2, Ar, CO_2, and CO) have PAs less than 691 kJ mol^{-1} and, thus are not detected by PTR-ToF-MS. Most VOCs have PAs greater than water and can be detected, with the exception of alkanes smaller than C5.[3] Though energy upon collision of VOCs with H_3O^+ is mild enough to leave most molecules intact, fragmentation often occurs for weak chemical bonds and/or large exothermicity of proton-transfer-reactions.

Appendix A.2. PTR-ToF-MS Experimental Settings and Tedlar® Bags

Prior to each participant visit, mass-to-charge calibration was performed using four isotopic ions commonly observed in room air: *m/z* 21.0221 ($H_3{}^{18}O^+$), 33.9935 ($^{18}O^{16}O^+$), 39.0327 ($H_2{}^{18}O)H_3{}^{16}O^+$), and 59.0491 $[C_3H_6O + H]^+$. The PTR-ToF-MS drift tube settings for temperature, pressure, and voltage were 60 °C (T_{drift}), 2.29 mbar (p_{drift}), and 600 V (U_{drift}), respectively, resulting in a ratio of the electric field (E) to the number density of the drift tube buffer gas molecules (N) of E/N ~132 Td (where Td = 10^{17} V cm^2). Analyte sampling was conducted at a flow rate of 0.1 L min^{-1} through a PEEK tubing inlet (1.59 mm O.D.) heated to 70 °C. The data were acquired with a 1 s time resolution. Depending on the species measured, the limit of detection was in the range of several parts per billion by volume (ppbv) or better. PTR-ToF-MS calibration was conducted using a standard solution of VOCs (acetone, formic acid, acetic acid, acetaldehyde, and formaldehyde) at known concentrations (5, 10, 30, 50, 100, 150, 200, 250 ppbv). Through analysis of the calibration curves (**Figure S1**), it was determined that small acids were approximately 30% underestimated by the PTR-ToF-MS, ketones were approximately 10% overestimated, and aldehydes had varied results, acetaldehyde was correctly determined by PTR-ToF-MS but formaldehyde was 64% underestimated. These correction values were not applied to participant data due to much larger fragmentation pattern discrepancies discussed in the manuscript.

During each participant visit, a Tedlar® bag was connected to the PTR-ToF-MS inlet for participant sampling. The most common material of Tedlar® bags is polyvinylfluoride (PVF) $(C_2H_3F)_n$.[4] PTR-ToF-MS measurements of clean Tedlar® bags show large peaks at *m/z* 88.0757 (N,N-dimethylacetamide, $C_4H_{10}NO^+$) and *m/z* 95.0491 (phenol, $C_6H_7O^+$). Both compounds are known to be present in Tedlar® bag production and have been seen in the gas-phase measurements of previous studies.[4–8] These compounds are quantitatively excluded from our results, as the background levels remain constant throughout sampling. Tedlar® bags were cleaned 3x prior to

each participant sampling through purging gas-phase pollutants by evacuating the bags and refilling them with clean air from a purge air generator (filtered and scrubbed of VOCs, CO_2, and water vapor). This cleaning did not fully remove "sticky", residual compounds that were adsorbed on bag walls (i.e., nicotine). Removing residual compounds would require heating the bags to 60 °C and evacuating/filling them with clean air multiple times, which was not feasible within the turnaround time between all participants.[4] Nicotine (*m/z* 163.1223. $C_{10}H_{15}N_2^+$) was present in the background prior to sampling but background levels did not affect the increases observed from new vapes into the bag.

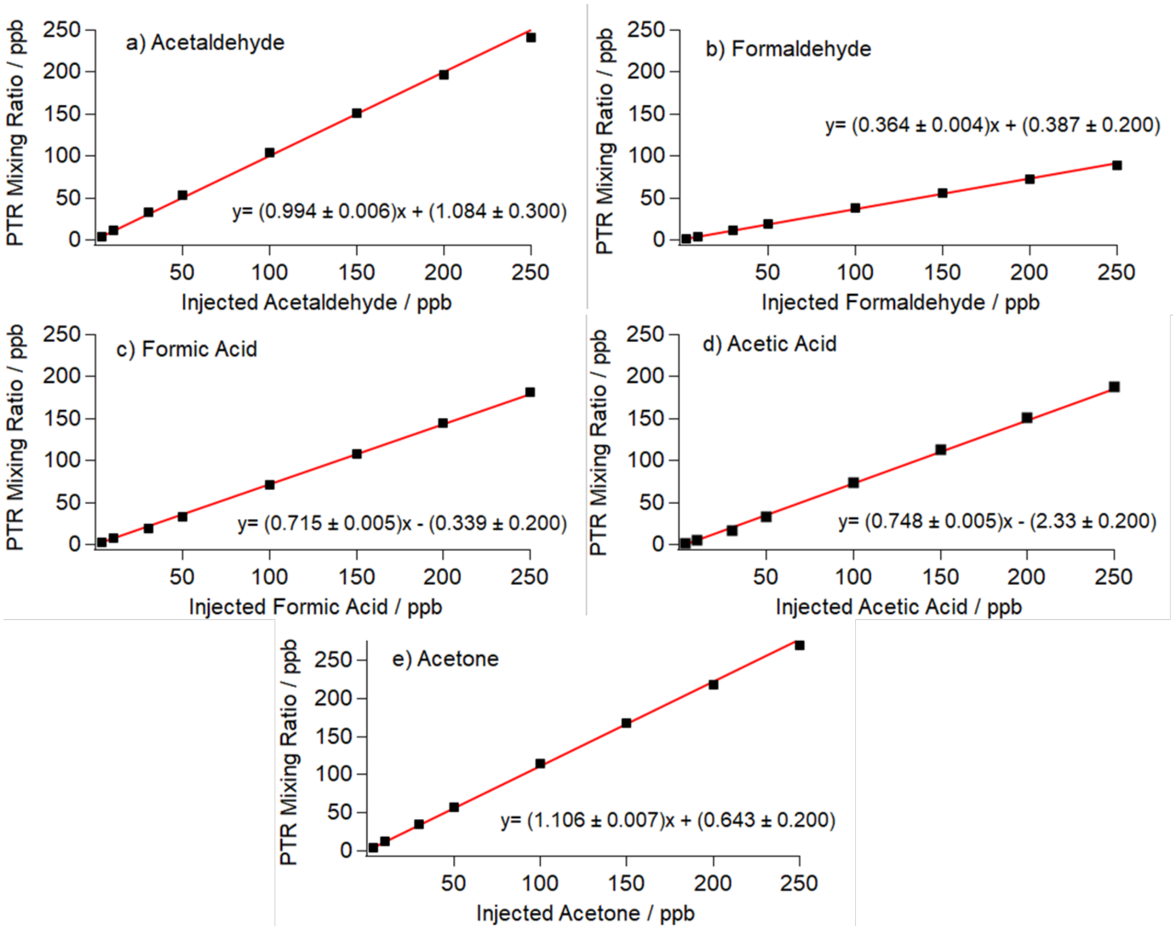

Figure S1. PTR-ToF-MS calibration was conducted using standard solutions of VOCs: a) acetaldehyde, b) formaldehyde, c) formic acid, d) acetic acid, and e) acetone at known concentrations (5, 10, 30, 50, 100, 150, 200, 250 ppbv). Through analysis of these calibration curves, it was determined that small acids were approximately 30% underestimated by the PTR-ToF-MS, ketones were approximately 10% overestimated, and aldehydes had varied results, acetaldehyde was correctly determined by PTR-ToF-MS but formaldehyde was 64% underestimated. These trends were not factored into our reported concentrations, as our uncertainty in this human trial study far exceeds these errors.

Appendix A.3. Programmable Pump for Simulated Puffing (Direct Injection Measurements)

A customized programable syringe pump was manufactured to accommodate a Tomopal 50 mL glass syringe, facilitating the delivery of 20 mL of e-cigarette aerosol into a Tedlar® bag. Utilizing computer numerical control (CNC) machining, a syringe pump chassis was created from a 15.24 x 30.48 x 5.08 cm white ultra-high molecular weight polyethylene sheet. This chassis included two linear motion carbon steel shafts measuring 9.52 mm in diameter and 228.60 mm in length on each side, equipped with bushings to reduce friction during operation. Positioned between the linear motion shafts, a stepper motor connected to a threaded shaft measuring 300 mm in length was utilized to regulate the syringe pump's movement. The stepper motor was interfaced with a Raspberry Pi 3 model B+, which controlled the stepper motor through the general-purpose input/output (GPIO) pins using the Python programming language. The 20 mL aspiration and extraction processes of the syringe were represented by the step angle of the stepper motor, whereas the flow rate was determined by the delay value, which represents the delay between each step of the stepper motor.

Appendix A.4. PTR-ToF-MS Data Analysis

A peak list was generated from ions observed in the mass spectra of participant samples. Ions were identified by comparison with the publicly available library (www.tinyurl.com/ PTRLibrary).[9] This library contains 1000 trace gases detected by PTR-MS and displays the fragmentation patterns of compounds after proton transfer reactions. Once a peak list was compiled, a ratio of each ion's intensity in spectrum A (exhaled puff) to spectrum B (background breath) was taken. Ions in the peak list with ratio > 1 were focused on for further analysis.

After injection of each sample (baseline breath, exhaled vape puff, or direct vape injection) into the Tedlar® bag, the signal plateaued and stabilized. For each VOC, an average of the signal was taken over 5 minutes after stabilization. This average signal was converted to mixing ratio of the corresponding gas. The PTR-MS Viewer software uses **Equation S1** to convert average signal intensity into mixing ratio (C^i_{ppb}) in the unit of ppbv.

$$C^i_{ppbv} = \frac{10^9}{k} \times \frac{22400 \cdot \mu_0 \cdot U_{drift}}{N_A \cdot l^2} \times \frac{T^2_{drift}}{T^2_0} \times \frac{p^2_0}{p^2_{drift}} \times \frac{TR_{H3O+}}{C_{H3O+}} \times \sum_i \frac{C^+_i \cdot Iso_i}{TR_i} \qquad \textbf{Equation S1}$$

Other terms in **Equation S1** are defined as:

- k = ion-molecule collision reaction rate constant (= 2.0 x 10^{-9} cm^3s^{-1}). While this rate constant can range from 1.5 to ~5 x 10^{-9} cm^3s^{-1}, a single value for the rate constant was used for all VOC.[3,9] This variable contributes the largest to the uncertainty in the determination of VOC estimated concentrations, which is expected to be ± 50%.
- μ_0 = the reduced ion mobility (= 2.8 cm^2 V^{-1} s^{-1})
- U_{drift} = the total voltage across the drift tube (= 600 V)
- N_A = the Avogadro number
- l = the length of the drift tube (= 9.3 cm)
- T_{drift} = the drift tube temperature (= 333 K)

- T_0 = the temperature at standard conditions (= 273.15 K)
- p_{drift} = the drift tube pressure (= 2.29 mbar)
- p_0 = the pressure at standard conditions (= 1013.25 mbar)
- C_i^+ = the measured signal (in cps) for the $[M+H]^+$ ion of interest
- C_{H3O+} = the measured signal (in cps) of the reagent ion (signal at *m/z* 21 was used (× 500), which is the isotopic ratio for $H_3O^+/H_3{}^{18}O^+$, due to *m/z* 19 signal being too large to quantify)
- TR_i = the transmission efficiency of the ion of interest (from Ionicon Analytik, see below). Transmission factors (TR_i) were measured regularly using a certified cylinder containing 14 aromatic compounds (Restek, TO-14A mix) ranging from benzene (*m/z* 79.0448) to 1,2,4-trichlorobenzene (*m/z* 180.9379). Some uncertainty is associated with this determination and the transmission used in this study are given below.
    - *m/z* 19 (TR_i= 0.18)
    - *m/z* 93 (TR_i= 0.57)
    - *m/z* 105 (TR_i= 0.66)
    - *m/z* 113 (TR_i= 0.71)
    - *m/z* 121 (TR_i= 0.74)
    - *m/z* 147 (TR_i= 0.85)
    - *m/z* 181 (TR_i= 1)
- TR_{H3O+} = the transmission efficiency of H_3O^+ *m/z* 19 (= 0.18)
- Iso_i = the isotopic abundance of the ion of interest.

Appendix A.5. Calculations of mass concentrations (µg VOC puff^{-1}) from concentrations (ppbv) generated by PTR-Viewer Software

Concentration values (ppbv) determined by the PTR-Viewer Software were converted into mass concentrations (µg VOC puff^{-1}) using **Equations S2-6**. $\Delta ppbv_{Baseline}$ is the concentration of baseline VOC subtracted from the clean air inside the bag (**Equation S2**). $\Delta ppbv_{Exhaled}$ is the concentration of VOC in the exhaled puff subtracted from the background breath concentration (**Equation S3**). $\Delta ppbv_{Direct\ Injection}$ is the concentration of VOC in the direct injection subtracted from the exhaled puff VOC concentration, as the Tedlar® bag was not cleaned after exhaled puff and the PTR-ToF-MS signal is additive (**Equation S4**). For the baseline breath, exhaled puff (after e-cigarette inhalation), and direct injection of e-cigarette aerosol, $\Delta ppbv$ for each VOC was converted into µg VOC in the bag using **Equation S5** and the following: concentration of air molecules under ambient conditions (2.46 x 10^{19} molecules cm^{-3}), the molecular weight of each VOC, and the volume of clean air inside the Tedlar® bag (80 L).

$$\Delta ppbv_{Baseline} = ppbv_{Baseline} - ppbv_{Clean\ air}$$ **Equation S2**

$$\Delta ppbv_{Exhaled} = ppbv_{Exhaled} - \Delta ppbv_{Baseline}$$ **Equation S3**

$$\Delta ppbv_{Direct\ Injection} = ppbv_{Direct\ Injection} - \Delta ppbv_{Exhaled}$$ **Equation S4**

$$\mu g\ VOC\ puff^{-1} = \Delta ppbv_{Baseline\ (or\ Exhaled\ or\ Direct\ Injection)}\ VOC\ \times\ 2.46\ \cdot 10^{10}\ \left(\frac{molecules}{ppbv\ cm^3}\right) \times \left(\frac{mol}{6.022x10^{23}\ molecules}\right)\ \times\ MW\ VOC\ \left(\frac{g}{mol}\right) \times\ 80\ L\ bag\ \times\ \left(\frac{1000\ cm^3}{1\ L}\right)\ \times\ \left(\frac{1\ \mu g}{10^{-6} g}\right)$$ **Equation S5**

In the case of the direct injection (**Equation S6**), there was no participant interaction with these measurements and thus needed to be corrected using the inhaled volume of each participant's puff (from topography measurements, **Table S1**) and the volume of direct injection (20 mL).

$$Corrected\ \mu g\ VOC\ puff^{-1} =\ \mu g\ VOC\ in\ the\ bag\ \times\ \left(\frac{V_{puff\ topography}\ (mL)}{V_{syringe}\ (mL)}\right)$$ **Equation S6**

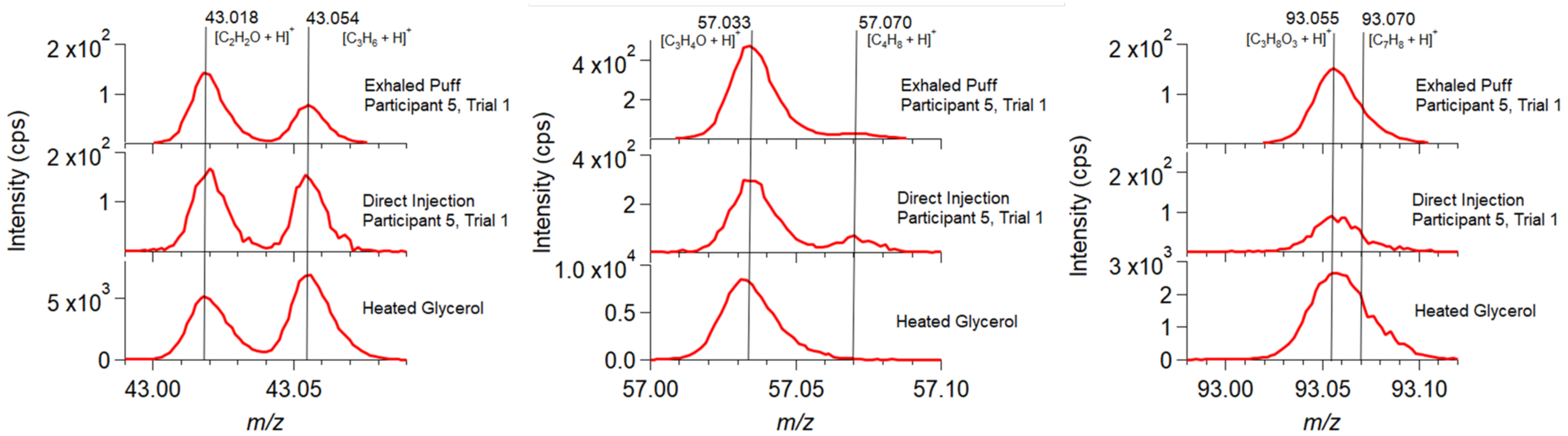

Figure S2. Expanded view of the PTR-ToF-MS spectra showing the separation of multiple peaks observed at nominal *m/z* 43, 57, and 93. Heated glycerol (bottom panel) is compared with exhaled puff (top) and direct injection (middle) measurements from participant 5's first visit.

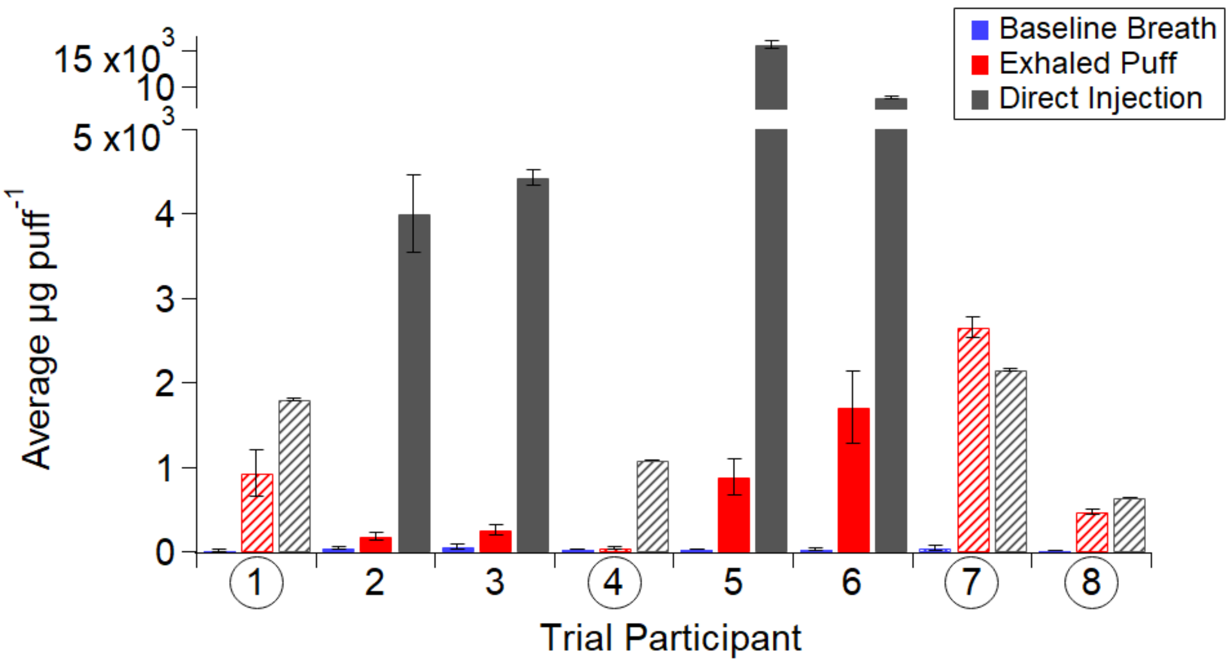

Figure S3. Liner scale of the combined mass concentrations of the species detectable by PTR-ToF-MS for all participant visits. The average mass per puff (µg VOC puff^{-1}) is shown for baseline breath (no vape, blue), exhaled puff (after inhaling from the vape device, red), and direct injection of vape aerosol (grey). The variability in the emissions is represented by error bars (one standard deviation) calculated using each participant's puff topography data (Table S1). Participants 1, 4, 7, and 8 used closed vapes (circled numbers, striped bars), whereas participants 2, 3, 5, and 6 used open vapes (solid bars). Note that this is the same plot as presented in the manuscript Figure 2, but in linear scale.

Appendix B. Whole Air Sampling Canisters and Gas Chromatography Systems Information

Appendix B.1. Whole Air Sampling Canisters Preparation and Sampling

Prior to collection, the canisters were first conditioned by baking them at 150 °C in humidified air for 12 h to form an oxidative layer inside the canisters that passivates the walls. Following the baking procedure, each canister was pumped-and-flushed several times using clean air to dilute any possible residual air inside the canisters (i.e., pressurization of the canisters to 40 psi followed by subsequent depressurization to ambient pressure), and then evacuated to 10^{-2} Torr. Finally, they were filled to 200 Torr with ultra-high purity helium and evacuated once again to 10^{-2} Torr. Each canister was fit with a Swagelok "T union" where the second end of the "T union" was connected with Teflon tubing (10 cm long). During sample collection, each subject exhaled through the Teflon tubing into the canister (while an operator manually opened the valve immediately after plugging the third end of the union) to allow the collection of their breath inside the canister.

Appendix B.2. Gas Chromatography Systems Information

Briefly, three GCs (all Hewlett-Packard HP-6890) and five column/detector combinations were used with flame ionization detection (FID), electron capture detection (ECD) and a mass selective detector (MSD) operating in selected ion monitoring (SIM) mode. The five column/detector combinations were DB-1 + FID, PLOT/DB-1 + FID, Restek-1701 + ECD, Restek-1701/DB-5 + ECD, and DB-5ms + MSD. To analyze a WAS sample, the canister was connected to the analytical system and 260 cm^{-3} of sample was pre-concentrated with liquid nitrogen via a loop which is filled with glass beads to increase surface area upon which the compounds of interest can condense. After the pre-concentration step, the VOCs were re-volatilized by replacing the liquid nitrogen with near boiling water (84 ºC) and then the VOCs were injected with a helium carrier to a splitter box that separated the sample flow into five streams, each directed to one of the column/detectors.

Compounds eluted with highest precision on one or multiple column/detector combinations. When a high-precision peak eluted on two or more column/detector combinations the results were averaged (e.g., propane, propene, benzene, toluene, ethylbenzene, xylenes). The FIDs were used for hydrocarbon detection whereas the ECDs were used for the detection of halocarbons and alkyl nitrates. The mass spectrometer was used for sulfur and oxygenated species as well as a secondary (or sometimes tertiary) measurement of many hydrocarbons and halocarbons. The limit of detection (LOD), measurement precision and accuracy vary for each species. For the VOCs specifically discussed in this study, these parameters are summarized in **Table S2**.

Table S2. VOC limit of detection and measurement precision and accuracy for the GC platform.

Compound	LOD (ppt)	Precision (%)	Accuracy (%)
Propene	10	10	10
1,2-Propadiene	10	10	10
Toluene	10	10	10
Styrene	10	10	10
Isoprene	10	10	10
Monoterpene	10	20	20
Acetaldehyde	100	20	30
Acetone	100	20	30
Ethyl Acetate	50	20	30
Methyl butanoate	50	20	30
Methanol	100	20	30
Ethanol	100	20	30
Propanol	100	20	30
DMS	10	10	10

Appendix B.3. Calculations of mass concentrations (ug VOC puff-1) from concentrations (pptv) determined by the GC platform.

For the baseline breath and exhaled puff (after e-cigarette inhalation), pptv for each VOC was converted into µg VOC puff^{-1} using **Equation S7** and the following: concentration of air molecules under ambient conditions (2.46 x 10^{19} molecules cm^{-3}), the molecular weight of each VOC, and the volume of the WAS canister (2000 cm^3). Since the participants often did not exhale 2000 cm^3 worth of breath into the WAS can, a pressure correction was made by applying the ratio of the pressure inside of the can to 760 Torr.

$$\mu g\ VOC\ puff^{-1} = pptv_{Baseline\ (or\ Exhaled)}\ VOC\ \times\ 2.46\ \cdot 10^{7}\ \left(\frac{molecules}{pptv\ cm^{3}}\right) \times$$
$$\times\ \left(\frac{mol}{6.022x10^{23}\ molecules}\right)\ \times\ MW\ VOC\ \left(\frac{g}{mol}\right) \times\ \left(\frac{1000\ cm^{3}}{1\ L}\right)\ \times\ \left(\frac{1\ \mu g}{10^{-6} g}\right) \times 2000\ cm^{3}\ WAS\ can\ \times$$
$$\left(\frac{Pressure\ in\ can\ (Torr)}{760\ (Torr)}\right)$$
Equation S7

Table S3. Measured concentrations of a 13-component ambient air quality gas standard (Cylinder CC302254, 2000 psi, AiR Environmental Inc.). The concentrations in the rightmost column were recertified by the manufacturer in February 2024.

Compound	PTR-ToF-MS Concentration (ppm)[(a)]	GC Concentration (ppm)	Certified Concentration (ppm)
Acetaldehyde	1.9 (*m/z* 45)	0.56	0.72
Methanol	0.79 (*m/z* 33)	0.99	0.67
Ethanol[(b)(f)]	0.14 (*m/z* 47)	–	0.76
Acrolein	1.7 (*m/z* 57.033)	1.1	0.25
Propanal	–	0.90	0.11
Acetone	–	1.2	1.1
Propanal + Acetone[(c)]	3.2 (*m/z* 59)	n/a	n/a
2-Propanol[(d)]	1.7 (*m/z* 61, 43, 41)	0.80	0.67
Acetonitrile[(f)]	2.0 (*m/z* 42)	–	0.91
Methyl Tert-Butyl Ether (MTBE)[(f)]	–	–	1.1
MTBE + 1-butanol[(c)(e)]	0.99 (*m/z* 57.070)	n/a	n/a
Methacrolein (MAC)	–	0.77	0.73
Methyl Vinyl Ketone (MVK)[(f)]	–	–	0.24
MAC + MVK[(b)]	3.4 (*m/z* 71)	n/a	n/a
Methyl Ethyl Ketone	2.0 (*m/z* 73)	1.1	0.90
1-Butanol[(g)]	–	–	0.13

(a) No external calibration factor was applied for this analysis
(b) Inomata et al. (2009) reported that, ethanol fragments into H_3O^+ at higher E/N conditions, such as the ones used in this study (~132 Td). Consequently, the sensitivity at *m/z* 47 is reduced, consistent with our observation.
(c) The PTRMS cannot distinguish between isomers at *m/z* 59.0491 ($C_3H_7O^+$), 57.070 ($C_4H_9^+$), and 71.041 ($C_4H_6O^+$).
(d) Analysis of the headspace of the pure standard showed that 2-propanol heavily fragments into its $[M{+}H{-}H_2O]^+$ ion with an additional high intensity ion at *m/z* 41.040 ($C_3H_5^+$), accounting for 65% of the *m/z* 43 ($C_3H_7^+$) peak intensity.
(e) Both MTBE and 1-butanol do not show a $[M{+}H]^+$ parent peak at *m/z* 89 and 75 respectively; but instead, both heavily fragment into a major ion at *m/z* 57.070 ($C_4H_9^+$). Note that both compounds exhibit a small contribution to *m/z* 41.040 ($C_3H_5^+$) as well accounting for about 26% and 15% of the *m/z* 57 peak for MTBE and butanol respectively, which overlaps with 2-propanol fragment.
(f) Note that a few compounds co-elute on our GC-FID column, which is the detector used for quantification. If two co-eluting species were both present at high concentration, such as in the VOC mixture, their respective quantification could not be conducted. This was the case for ethanol and acetonitrile (co-eluted at 8.963 min), as well as MTBE and MVK (co-eluted at 11.298 min).
(g) 1-Butanol was not detected by GC.

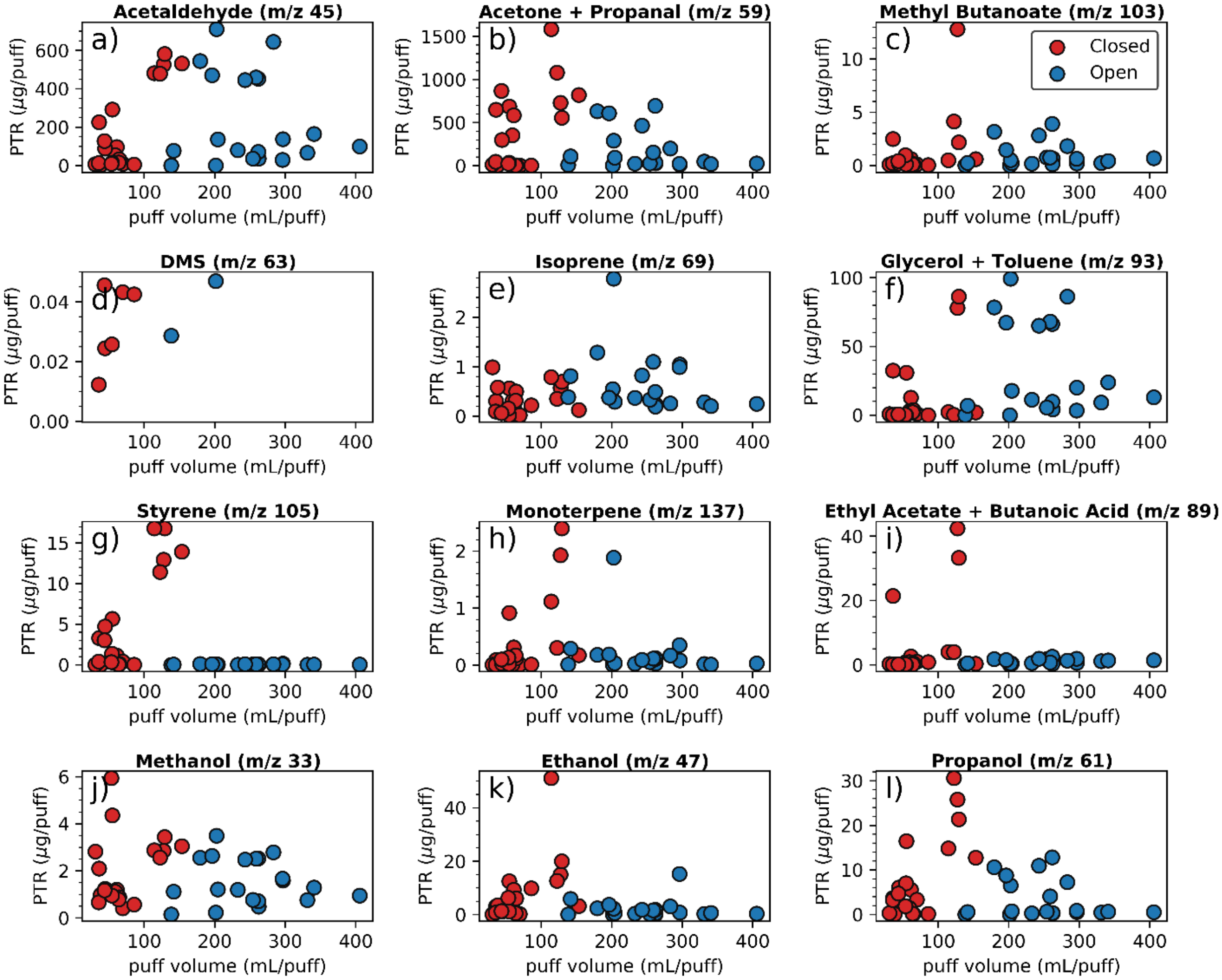

Figure S4. Exhaled puff PTR-ToF-MS values of micrograms per inhaled puff volume obtained from participant puff topography data. The puff volume reported is the volume each participant inhaled from their vape device prior to exhalation into the PTR-ToF-MS sampling unit. Twelve compounds were selected for this comparison. Red markers correspond to closed vapes and blue markers correspond to open vapes used in the human trial.

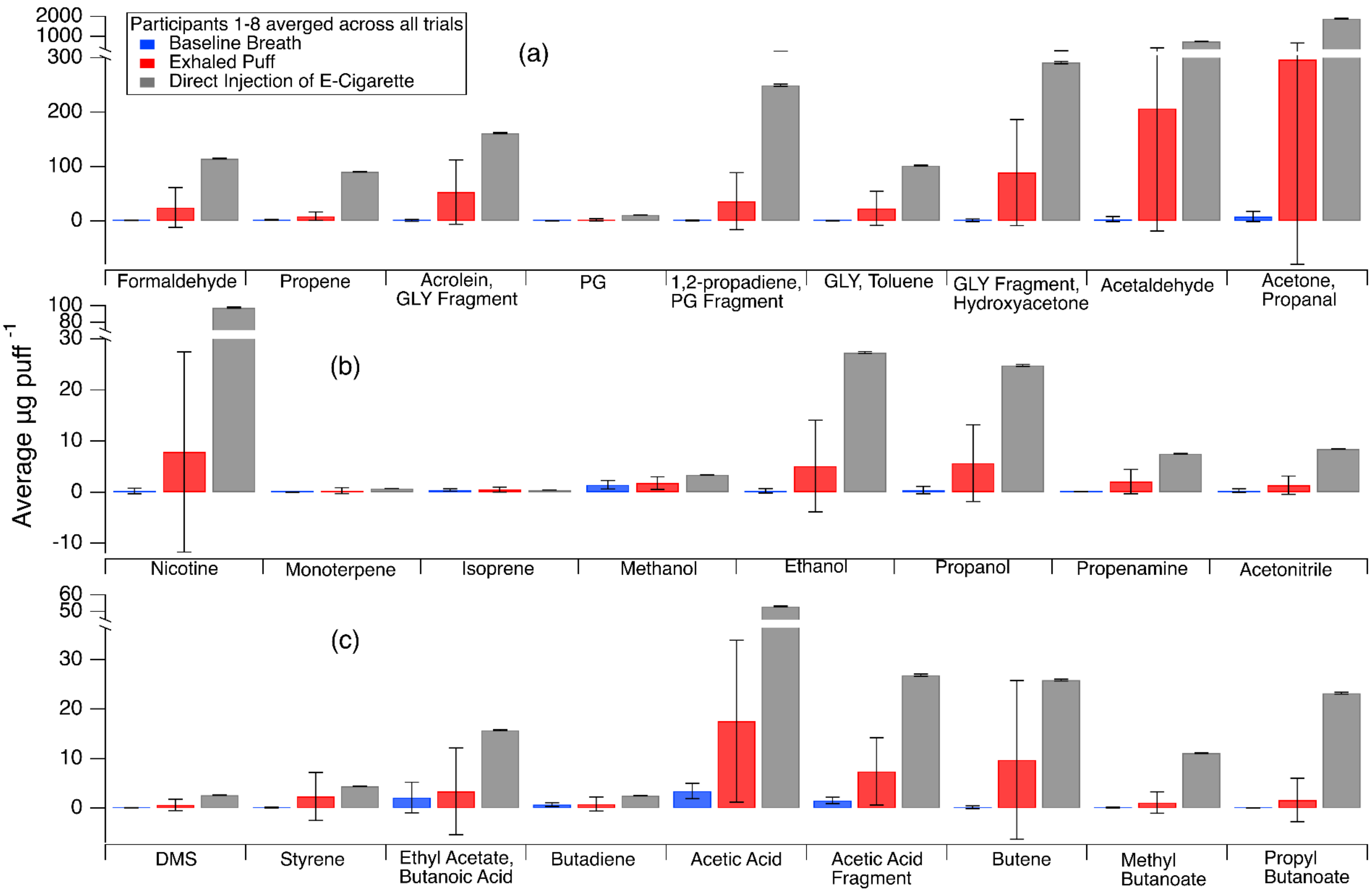

Figure S5. PTR-ToF-MS results of selected VOCs from all participant visits (40) with averaged measurements of baseline breath (no vape, blue), exhaled puff (after inhaling from the vape device, red), and direct injection of e-cigarette (grey). Error was calculated using each participant's puff topography data that varied by visit.

Table S4. Ions observed in the PTR-ToF-MS mass spectra (**Figures S5-12**) from participant exhaled vape puff measurements. Structural isomers cannot be distinguished using the PTR-ToF-MS, hence, multiple compound assignments are given for certain peaks.

**Nominal *m/z***	**Exact *m/z***	**Empirical Formula**	**Potential Assignment**	**Reference(s)**
**31**	31.0178	CH_3O^+	Formaldehyde	10
**33**	33.0335	CH_5O^+	Methanol	11
**41**	41.0386[(a)]	$C_3H_5^+$	1,2-Propadiene<br>Propylene glycol fragment (-2(H_2O))	12
**42**	42.0338	$C_2H_4N^+$	Acetonitrile	12
**43**	43.0178	$C_2H_3O^+$	Acetic acid fragment<br>Hydroxyacetaldehyde fragment<br>Acetate esters fragment<br>Glycolaldehyde fragment	13,14<br>13<br>15
	43.0542[(a)]	$C_3H_7^+$	Propene	11
**45**	45.0335	$C_2H_5O^+$	Acetaldehyde	10,16
**47**	47.0128	$CH_3O_2^+$	Formic acid	17,18
	47.0491	$C_2H_7O^+$	Ethanol	11,15,17
**55**	55.0542	$C_4H_7^+$	Butadiene	12
**57**	57.0335	$C_3H_5O^+$	Acrolein<br>Glycerol fragment (-2(H_2O))	12,13
	57.0699[(a)]	$C_4H_9^+$	Butene	19
**58**	58.0651	$C_3H_8N^+$	Propenamine	12
**59**	59.0491	$C_3H_7O^+$	Acetone/propanal<br>Propylene glycol fragment (-H_2O)	11,12,20
**61**	61.0284	$C_2H_5O_2^+$	Acetic acid, Acetate esters fragment<br>Hydroxyacetaldehyde<br>Glycolaldehyde	14<br>12
	61.0648	$C_3H_9O^+$	Propanol	15
**63**	63.0263	$C_2H_7S^+$	Dimethyl sulfide	12
**69**	69.0335	$C_4H_5O^+$	Furan	12,18
	69.0699	$C_5H_9^+$	Isoprene	11,21
**71**	71.0855[(a)]	$C_5H_{11}^+$	Pentene	19

**75**	75.0441	$C_3H_7O_2^+$	Hydroxyacetone<br>1-Hydroxypropanal<br>Propanoic acid<br>Glycerol fragment (-H_2O)	12<br>13<br>13<br>13
**77**	77.0597	$C_3H_9O_2^+$	Propylene glycol	22
**88**	88.0757	$C_4H_{10}NO^+$	N,N-Dimethylacetamide (DMAC)	Intrinsic impurity in Tedlar® bags 4,7
**89**	89.0597	$C_4H_9O_2^+$	Ethyl acetate<br>Butanoic acid	15<br>9,23
**91**	90.9477	$FeOH(H_2O)^+$	Intrinsic ion in PTR-ToF-MS source	13,24
	91.0390	$C_3H_7O_3^+$	Glyceraldehyde<br>Lactic acid	22<br>13
**93**	93.0546	$C_3H_9O_3^+$	Glycerol	13
	93.0699	$C_7H_9^+$	Toluene	11
**95**	95.0491	$C_6H_7O^+$	Phenol	Intrinsic impurity in Tedlar® bags 4,7
**103**	103.0754	$C_5H_{11}O_2^+$	Methyl butanoate	15
**105**	105.0699	$C_8H_9^+$	Styrene	12
**117**	117.0546	$C_5H_9O_3^+$	1-Acetoxyacetone	13,25
**123**	123.0804	$C_8H_{11}O^+$	2-Hydroxybenzaldehyde	12
**130**	130.1067	$C_7H_{15}O_2^+$	Propyl butanoate	15
**137**	137.1325	$C_{10}H_{17}^+$	Monoterpenes[(b)]	26
**163**	163.1230	$C_{10}H_{15}N_2^+$	Nicotine	13

[(a)] Alkanes, alkenes and aliphatic alcohols are known to fragment to small alkyl ions and will also contribute to m/z 41 ($C_3H_5^+$), 43 ($C_4H_7^+$), 57 ($C_4H_9^+$), 71 ($C_5H_{11}^+$)…etc.
[(b)] Monoterpenes are known to fragment into *m/z* 81 but due to the complexity of the samples, we excluded this ion from quantification.[27,28]

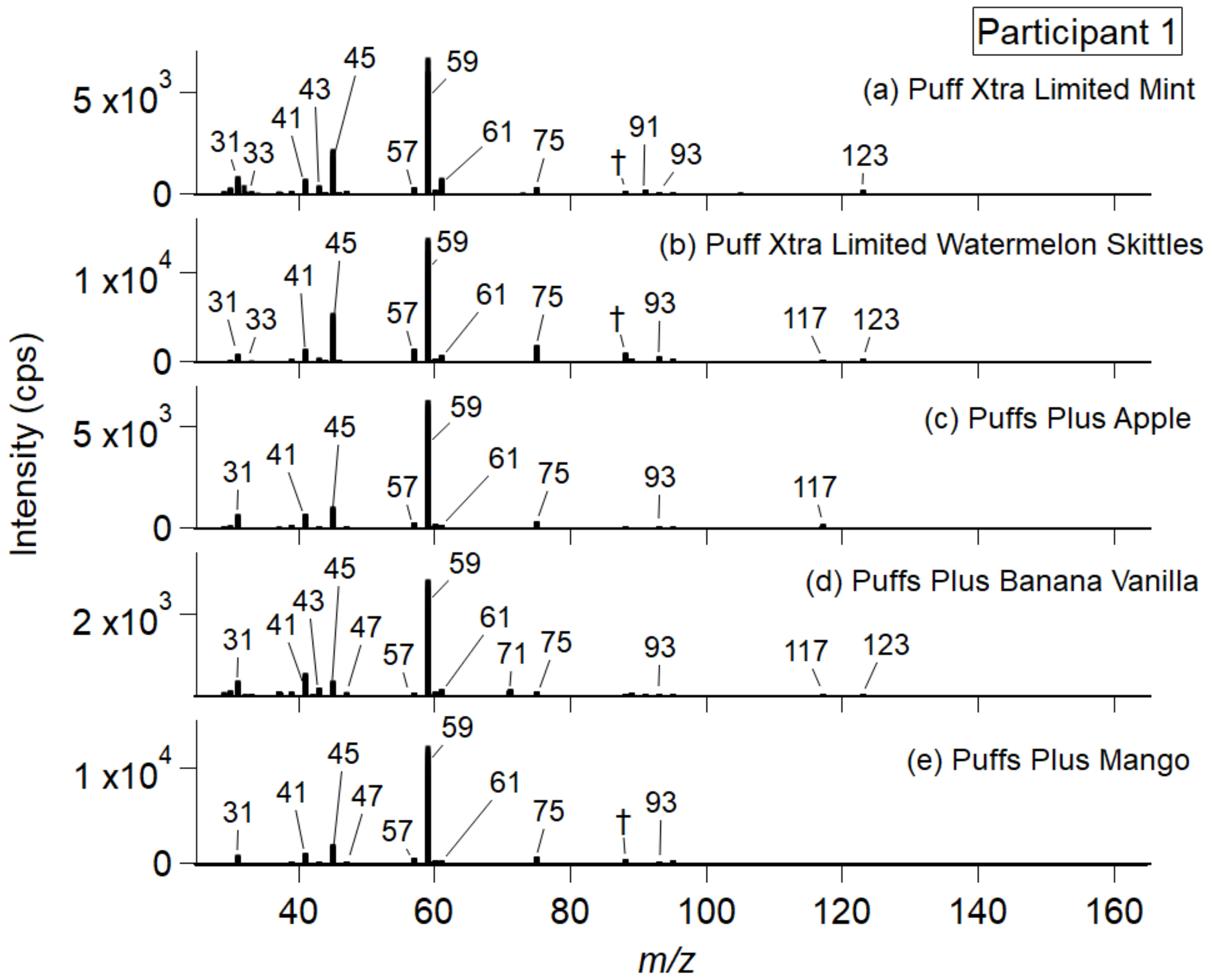

Figure S6. PTR-ToF-MS mass spectra for Participant 1's five visits using Puff Xtra Limited and Puffs Plus *closed* vapes in flavors of (a) mint, (b) watermelon skittles, (c) apple, (d) banana vanilla, and (e) mango. The presented spectra are of the exhaled puff measurements with background breath subtracted. The peak indicated with (†) at *m/z* 88 corresponds to DMAC, a known impurity of Tedlar® bags, which was incompletely removed by subtraction. Each spectrum corresponds to an average spectrum taken over 5 min.

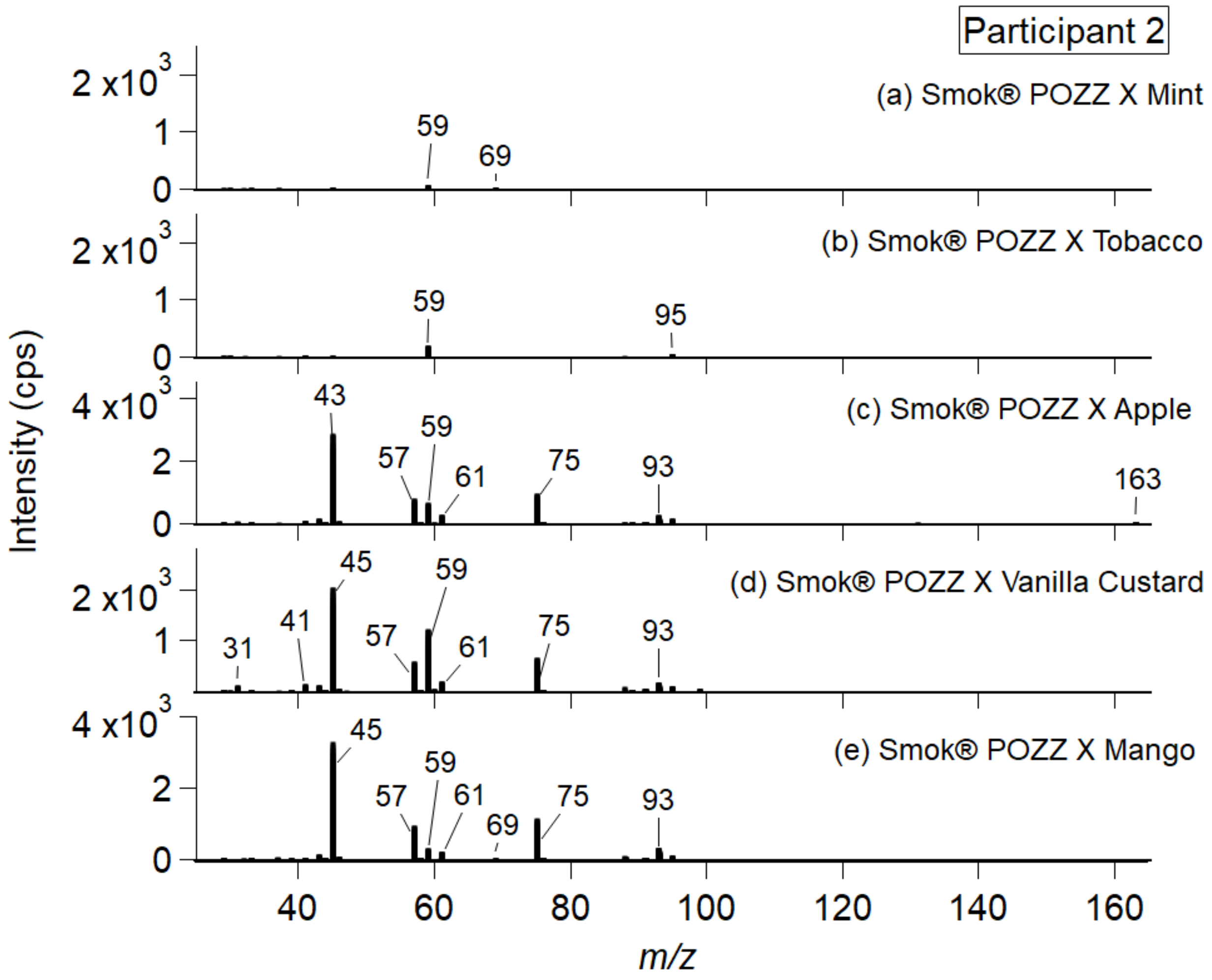

Figure S7. PTR-ToF-MS mass spectra for Participant 2's five visits using a Smok® POZZ X *open* vape with e-liquid in flavors of (a) mint, (b) tobacco, (c) apple, (d) vanilla custard, and (e) mango. The presented spectra are of the exhaled puff measurements with background breath subtracted. Each spectrum corresponds to an average spectrum taken over 5 min. It is important to note that this participant did not smoke watermelon (one of the 5 flavor profiles in this study). Tobacco was initially one of the five profiles selected for this study, but due to feedback from participants 2 and 3, it was discontinued and switched out for watermelon. Mass spectra (a) and (b) had very low signal and the dominant peaks were acetone and isoprene (common in breath without vape).

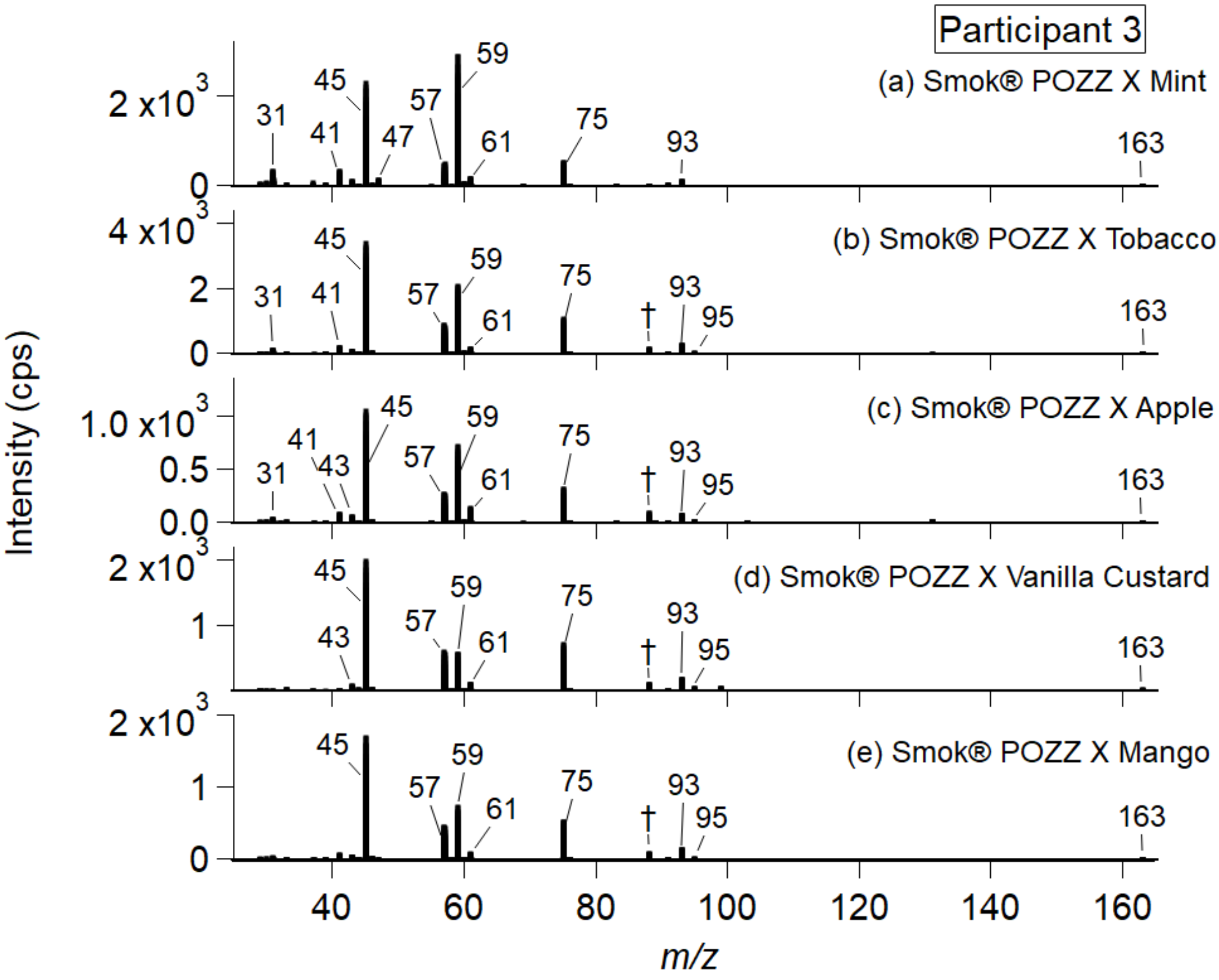

Figure S8. PTR-ToF-MS mass spectra for Participant 3's five visits using a Smok® POZZ X *open* vape with e-liquid in flavors of (a) mint, (b) tobacco, (c) apple, (d) vanilla custard, and (e) mango. The presented spectra are of the exhaled puff measurements with background breath subtracted. The peak indicated with (†) at *m/z* 88 corresponds to DMAC, a known impurity of Tedlar® bags, which was incompletely removed by subtraction. Each spectrum corresponds to an average spectrum taken over 5 min. It is important to note that this participant did not smoke watermelon (one of the 5 flavor profiles in this study). Tobacco was initially one of the five profiles selected for this study, but due to feedback from participants 2 and 3, it was discontinued and switched out for watermelon.

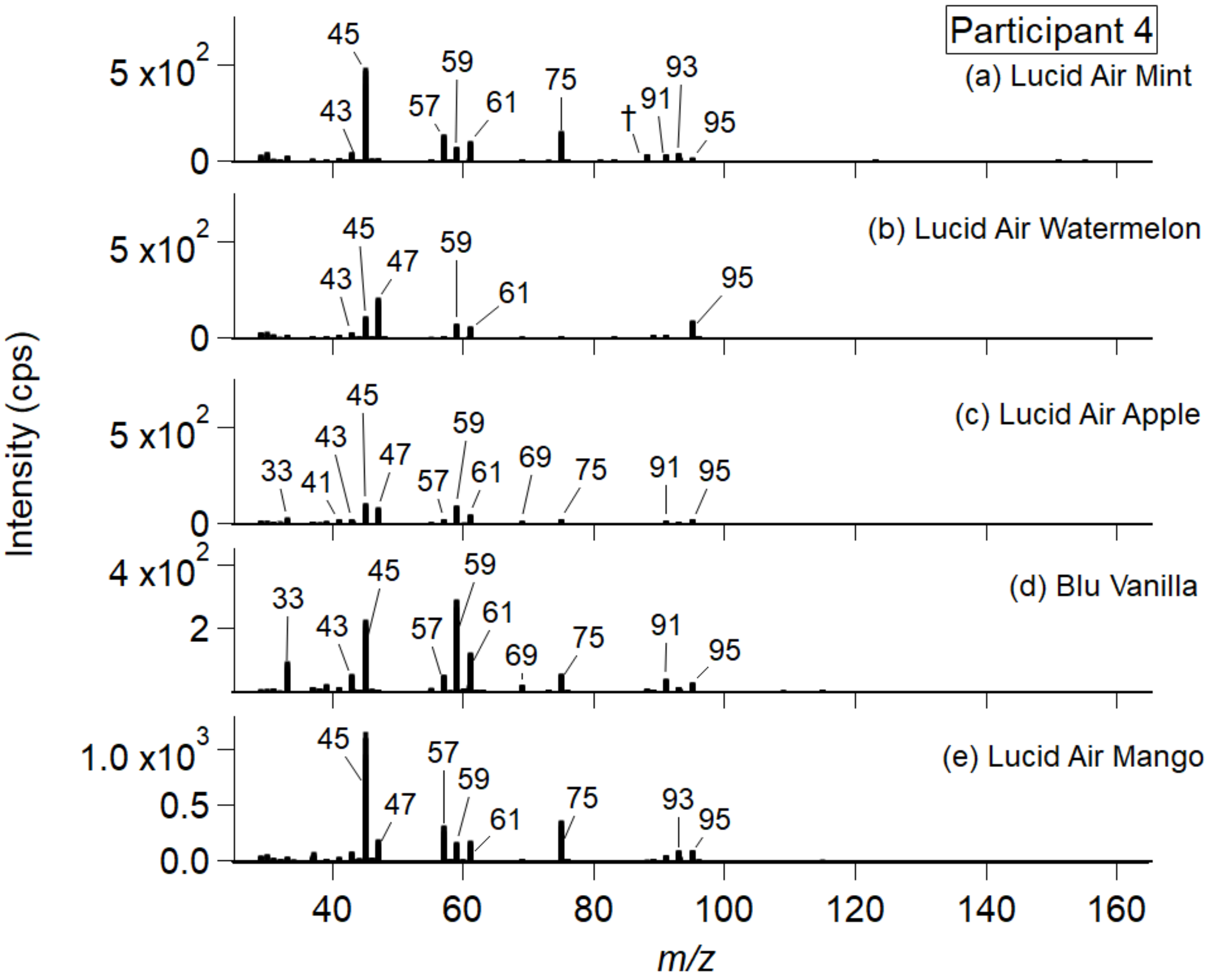

Figure S9. PTR-ToF-MS mass spectra for Participant 4's five visits using Lucid Air and Blu *closed* vapes in flavors of (a) mint, (b) watermelon, (c) apple, (d) vanilla, and (e) mango. The presented spectra are of the exhaled puff measurements with background breath subtracted. The peak indicated with (†) at *m/z* 88 corresponds to DMAC, a known impurity of Tedlar® bags, which was incompletely removed by subtraction. Each spectrum corresponds to an average spectrum taken over 5 min.

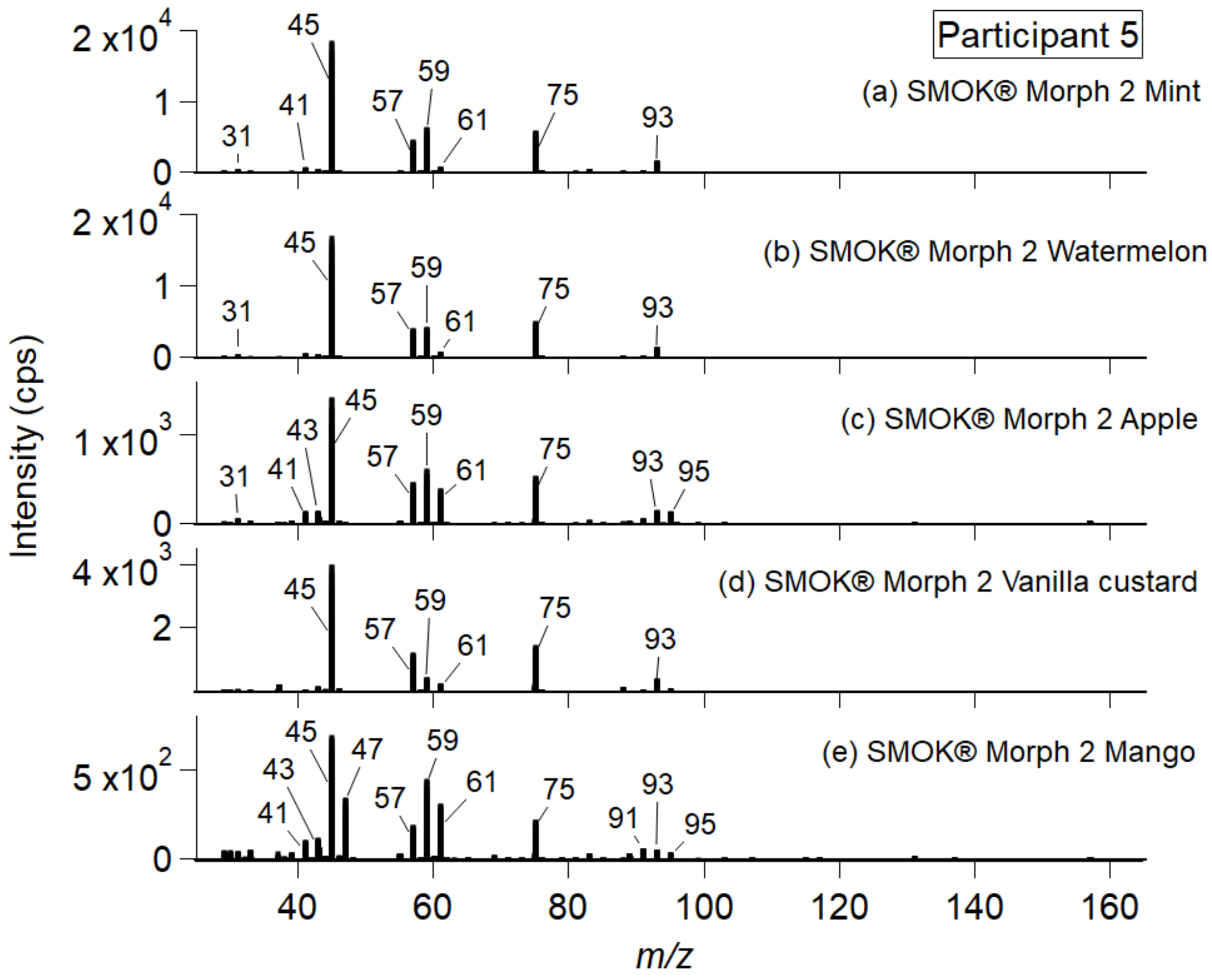

Figure S10. PTR-ToF-MS mass spectra for Participant 5's five visits using a SMOK® Morph 2 *open* vape with e-liquid flavors of (a) mint, (b) watermelon, (c) apple, (d) vanilla custard, and (e) mango. The presented spectra are of the exhaled puff measurements with background breath subtracted. Each spectrum corresponds to an average spectrum taken over 5 min.

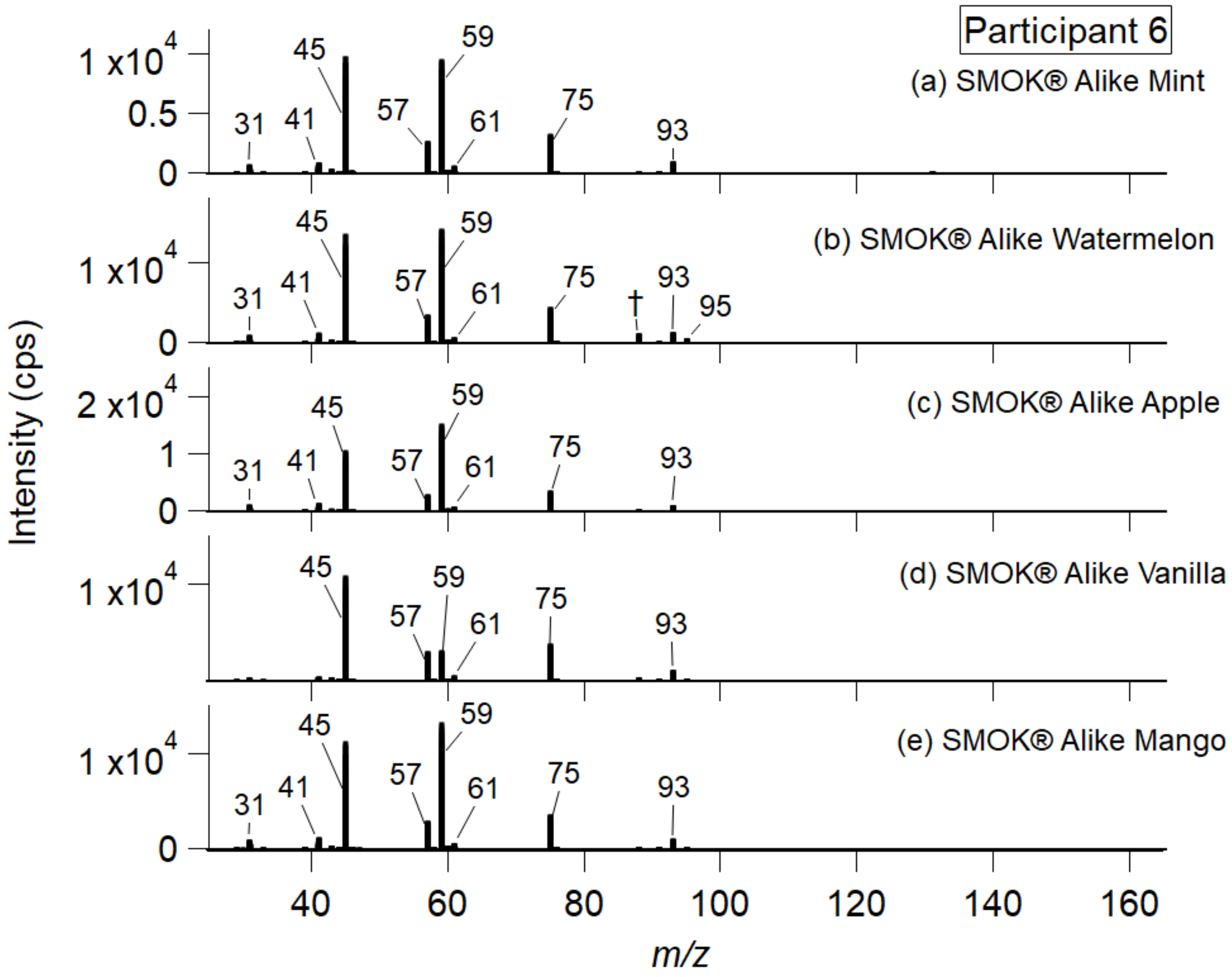

Figure S11. PTR-ToF-MS mass spectra for Participant 6's five visits using a SMOK® Alike *open* vape with e-liquid flavors of (a) mint, (b) watermelon, (c) apple, (d) vanilla, and (e) mango. The presented spectra are of the exhaled puff measurements with background breath subtracted. The peak indicated with (†) at *m/z* 88 corresponds to DMAC, a known impurity of Tedlar® bags, which was incompletely removed by subtraction. Each spectrum corresponds to an average spectrum taken over 5 min.

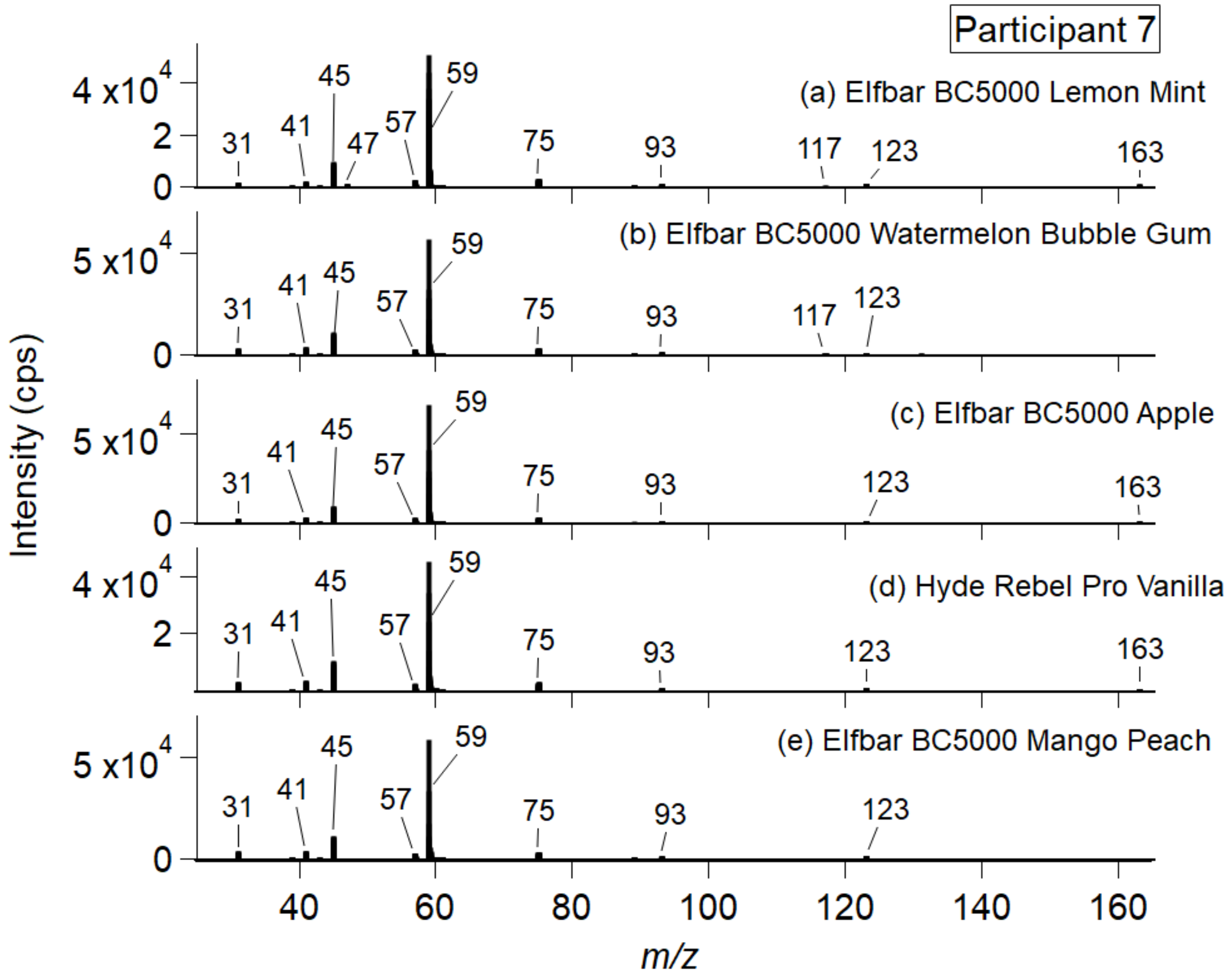

Figure S12. PTR-ToF-MS mass spectra for Participant 7's five visits using Elfbar BC5000 and Hyde Rebel Pro *closed* vapes with e-liquid flavors of (a) lemon mint, (b) watermelon bubble gum, (c) apple, (d) vanilla, and (e) mango peach. The presented spectra are of the exhaled puff measurements with background breath subtracted. Each spectrum corresponds to an average spectrum taken over 5 min.

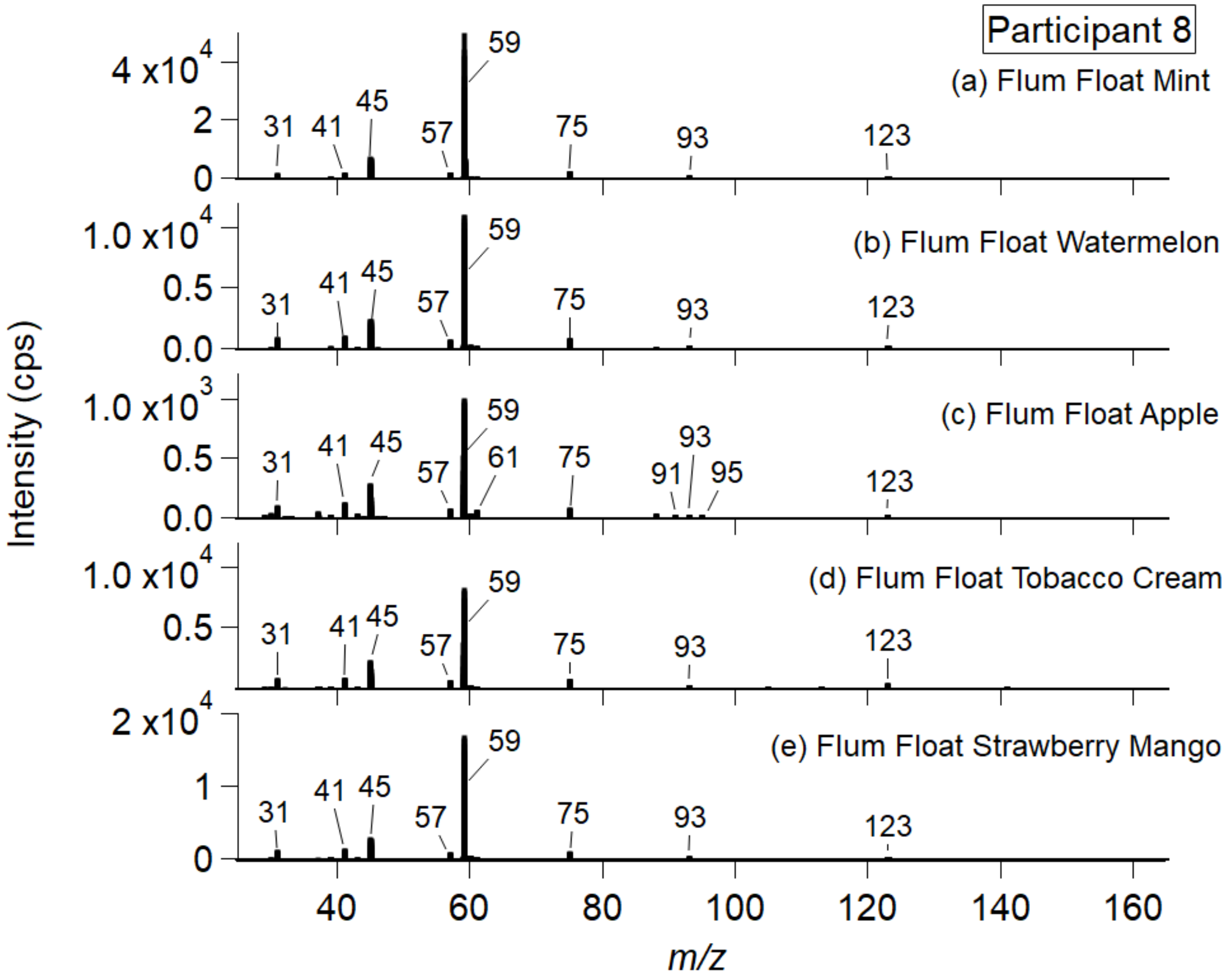

Figure S13. PTR-ToF-MS mass spectra for Participant 8's five visits using Flum Float *closed* vapes with e-liquid flavors of (a) mint, (b) watermelon, (c) apple, (d) tobacco cream, and (e) strawberry mango. The presented spectra are of the exhaled puff measurements with background breath subtracted. Each spectrum corresponds to an average spectrum taken over 5 min. Tobacco cream is Flum Float's equivalent product to vanilla that other participants smoked.

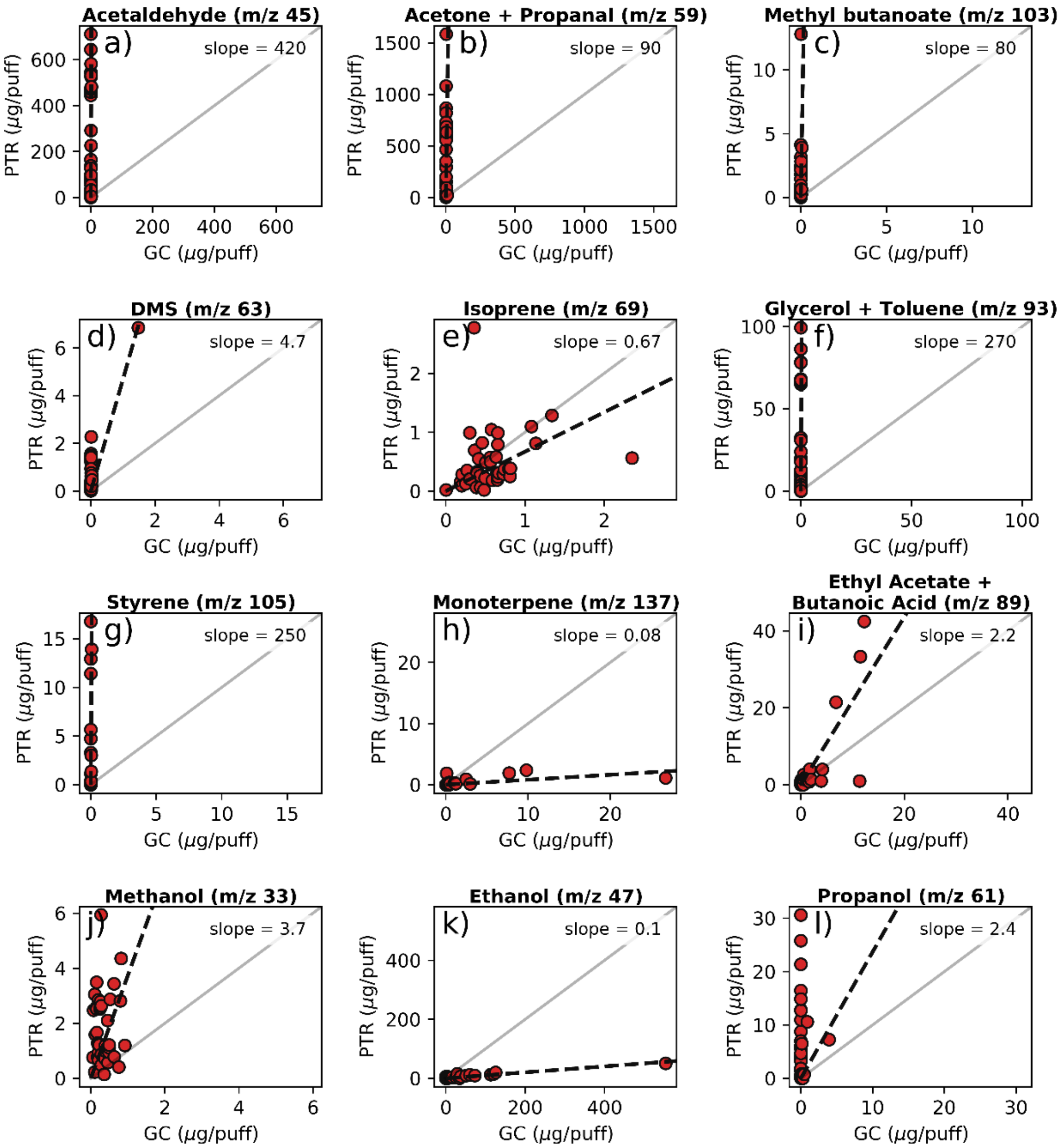

Figure S14. Scattered data for each PTR/GC exhaled breath measurement, in calculated micrograms per puff. Black dashed lines are the linear fit for participant data. Diagonal 1:1 lines (solid grey) illustrate the lack of correlation between measurements. All signal intensities reported for the PTR-ToF-MS correspond to the $[M+H]^+$ ion of each of the reported species. In panel (b) while acetone is presented exclusively for the GC measurements, the PTRMS signal is for the sum of acetone and propanal (*m/z* 59, $C_3H_7O^+$). In panel (f), due to the overwhelming presence of the humectant, the PTR-ToF-MS signal is given for the sum of GLY and toluene, while the GC measurements are reported for toluene exclusively. In panel (i), while ethyl acetate is presented exclusively for the GC measurements, the contribution of butanoic acid couldn't be excluded for the PTR-ToF-MS and the signal is for the sum of ethyl acetate and butanoic acid. Note that the slopes are included for reference only to demonstrate how the two measurements deviate from one another, not to suggest that there is correlation between the two datasets.

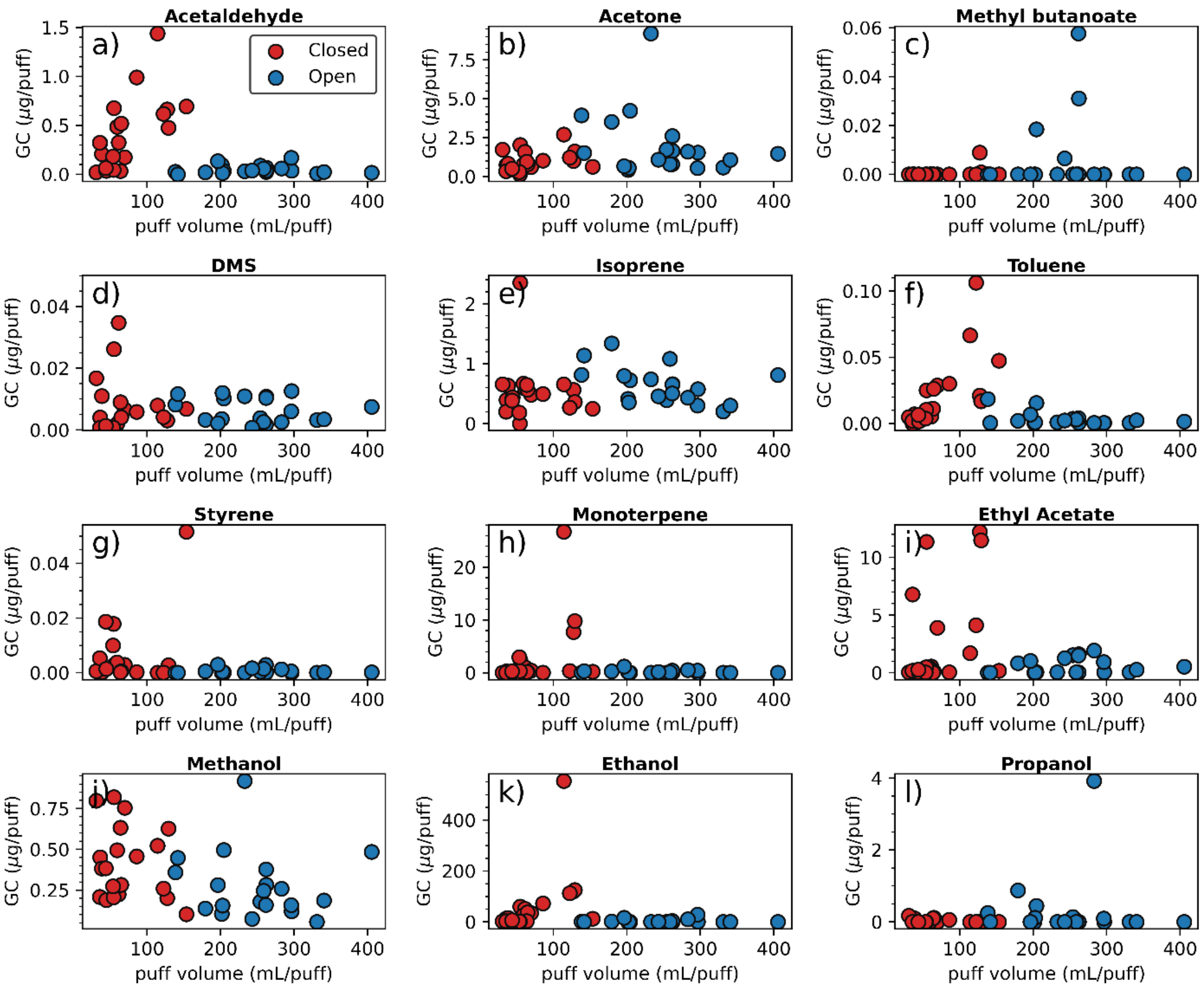

Figure S15. Exhaled puff GC values of micrograms per puff with respect to participant puff topography data. Twelve compounds were selected for this comparison. Red markers correspond to *closed* vapes and blue markers correspond to *open* vapes used in the human trial.

References

(1) Pagano, T.; Bida, M. R.; Robinson, R. J. Laboratory Activity for the Determination of Nicotine in Electronic Cigarette Liquids Using Gas Chromatography-Mass Spectrometry. *Journal of Laboratory Chemical Education* **2015**, *3* (3), 37–43.

(2) Hunter, E. P. L.; Lias, S. G. Evaluated Gas Phase Basicities and Proton Affinities of Molecules: An Update. *Journal of Physical and Chemical Reference Data* **1998**, *27* (3), 413–656. https://doi.org/10.1063/1.556018.

(3) Zhao, J.; Zhang, R. Proton Transfer Reaction Rate Constants between Hydronium Ion (H3O+) and Volatile Organic Compounds. *Atmospheric Environment* **2004**, *38* (14), 2177–2185. https://doi.org/10.1016/j.atmosenv.2004.01.019.

(4) Beauchamp, J.; Herbig, J.; Gutmann, R.; Hansel, A. On the Use of Tedlar® Bags for Breath-Gas Sampling and Analysis. *Journal of Breath Research* **2008**, *2* (4), 046001. https://doi.org/10.1088/1752-7155/2/4/046001.

(5) Jacek A. Koziel; Jarett P. Spinhirne; Jenny D. Lloyd; David B. Parker; Donald W. Wright; Fred W. Kuhrt. Evaluation of Sample Recovery of Malodorous Gases from Air Sampling Bags, SPME, and Sampling Canisters. In *2004, Ottawa, Canada August 1 - 4, 2004*; American Society of Agricultural and Biological Engineers, 2004. https://doi.org/10.13031/2013.16814.

(6) Trabue, S. L.; Anhalt, J. C.; Zahn, J. A. Bias of Tedlar Bags in the Measurement of Agricultural Odorants. *Journal of Environmental Quality* **2006**, *35* (5), 1668–1677. https://doi.org/10.2134/jeq2005.0370.

(7) Mochalski, P.; King, J.; Unterkofler, K.; Amann, A. Stability of Selected Volatile Breath Constituents in Tedlar, Kynar and Flexfilm Sampling Bags. *Analyst* **2013**, *138* (5), 1405–1418. https://doi.org/10.1039/C2AN36193K.

(8) Pet'ka, J.; Étievant, P.; Callement, G. Suitability of Different Plastic Materials for Head or Nose Spaces Short Term Storage. *Analusis* **2000**, *28* (4), 330–335. https://doi.org/10.1051/analusis:2000123.

(9) Pagonis, D.; Sekimoto, K.; de Gouw, J. A Library of Proton-Transfer Reactions of H_3O^+ Ions Used for Trace Gas Detection. *Journal of the American Society for Mass Spectrometry* **2019**, *30* (7), 1330–1335. https://doi.org/10.1007/s13361-019-02209-3.

(10) Inomata, S.; Tanimoto, H.; Kameyama, S.; Tsunogai, U.; Irie, H.; Kanaya, Y.; Wang, Z. Technical Note: Determination of Formaldehyde Mixing Ratios in Air with PTR-MS: Laboratory Experiments and Field Measurements. *Atmospheric Chemistry and Physics* **2008**, *8* (2), 273–284.

(11) Inomata, S.; Tanimoto, H.; Kato, S.; Suthawaree, J.; Kanaya, Y.; Pochanart, P.; Liu, Y.; Wang, Z. PTR-MS Measurements of Non-Methane Volatile Organic Compounds during an Intensive Field Campaign at the Summit of Mount Tai, China, in June 2006. *Atmospheric Chemistry and Physics* **2010**, *10* (15), 7085–7099. https://doi.org/10.5194/acp-10-7085-2010.

(12) Koss, A. R.; Sekimoto, K.; Gilman, J. B.; Selimovic, V.; Coggon, M. M.; Zarzana, K. J.; Yuan, B.; Lerner, B. M.; Brown, S. S.; Jimenez, J. L.; Krechmer, J.; Roberts, J. M.; Warneke, C.; Yokelson, R. J.; de Gouw, J. Non-Methane Organic Gas Emissions from Biomass Burning: Identification, Quantification, and Emission Factors from PTR-ToF during the FIREX 2016 Laboratory Experiment. *Atmospheric Chemistry and Physics* **2018**, *18* (5), 3299–3319. https://doi.org/10.5194/acp-18-3299-2018.

(13) Perraud, V.; Lawler, M. J.; Malecha, K. T.; Johnson, R. M.; Herman, D. A.; Staimer, N.; Kleinman, M. T.; Nizkorodov, S. A.; Smith, J. N. Chemical Characterization of Nanoparticles and Volatiles Present in Mainstream Hookah Smoke. *Aerosol Science and Technology* **2019**, *53* (9), 1023–1039. https://doi.org/10.1080/02786826.2019.1628342.

(14) Haase, K. B.; Keene, W. C.; Pszenny, A. a. P.; Mayne, H. R.; Talbot, R. W.; Sive, B. C. Calibration and Intercomparison of Acetic Acid Measurements Using Proton-Transfer-Reaction Mass Spectrometry (PTR-MS). *Atmospheric Measurement Techniques* **2012**, *5* (11), 2739–2750. https://doi.org/10.5194/amt-5-2739-2012.

(15) Buhr, K.; van Ruth, S.; Delahunty, C. Analysis of Volatile Flavour Compounds by Proton Transfer Reaction-Mass Spectrometry: Fragmentation Patterns and Discrimination between Isobaric and Isomeric Compounds. *International Journal of Mass Spectrometry* **2002**, *221* (1), 1–7. https://doi.org/10.1016/S1387-3806(02)00896-5.

(16) Kajos, M. K.; Rantala, P.; Hill, M.; Hellén, H.; Aalto, J.; Patokoski, J.; Taipale, R.; Hoerger, C. C.; Reimann, S.; Ruuskanen, T. M.; Rinne, J.; Petäjä, T. Ambient Measurements of Aromatic and Oxidized VOCs by PTR-MS and GC-MS: Intercomparison between Four Instruments in a Boreal Forest in Finland. *Atmospheric Measurement Techniques* **2015**, *8* (10), 4453–4473. https://doi.org/10.5194/amt-8-4453-2015.

(17) Lindinger, W.; Hansel, A.; Jordan, A. On-Line Monitoring of Volatile Organic Compounds at Pptv Levels by Means of Proton-Transfer-Reaction Mass Spectrometry (PTR-MS) Medical Applications, Food Control and Environmental Research. *International Journal of Mass Spectrometry and Ion Processes* **1998**, *173* (3), 191–241. https://doi.org/10.1016/S0168-1176(97)00281-4.

(18) Warneke, C.; Roberts, J. M.; Veres, P.; Gilman, J.; Kuster, W. C.; Burling, I.; Yokelson, R.; de Gouw, J. A. VOC Identification and Inter-Comparison from Laboratory Biomass Burning Using PTR-MS and PIT-MS. *International Journal of Mass Spectrometry* **2011**, *303* (1), 6–14. https://doi.org/10.1016/j.ijms.2010.12.002.

(19) Gueneron, M.; Erickson, M. H.; VanderSchelden, G. S.; Jobson, B. T. PTR-MS Fragmentation Patterns of Gasoline Hydrocarbons. *International Journal of Mass Spectrometry* **2015**, *379*, 97–109. https://doi.org/10.1016/j.ijms.2015.01.001.

(20) Dunne, E.; Galbally, I. E.; Cheng, M.; Selleck, P.; Molloy, S. B.; Lawson, S. J. Comparison of VOC Measurements Made by PTR-MS, Adsorbent Tubes–GC-FID-MS and DNPH Derivatization–HPLC during the Sydney Particle Study, 2012: A Contribution to the Assessment of Uncertainty in Routine Atmospheric VOC Measurements. *Atmospheric Measurement Techniques* **2018**, *11* (1), 141–159. https://doi.org/10.5194/amt-11-141-2018.

(21) Brilli, F.; Gioli, B.; Ciccioli, P.; Zona, D.; Loreto, F.; Janssens, I. A.; Ceulemans, R. Proton Transfer Reaction Time-of-Flight Mass Spectrometric (PTR-TOF-MS) Determination of Volatile Organic Compounds (VOCs) Emitted from a Biomass Fire Developed under Stable Nocturnal Conditions. *Atmospheric Environment* **2014**, *97*, 54–67. https://doi.org/10.1016/j.atmosenv.2014.08.007.

(22) Španěl, P.; Wang, T.; Smith, D. A Selected Ion Flow Tube, SIFT, Study of the Reactions of H_3O^+, NO^+ and O_2^+ Ions with a Series of Diols. *International Journal of Mass Spectrometry* **2002**, *218* (3), 227–236. https://doi.org/10.1016/S1387-3806(02)00724-8.

(23) Henderson, B.; Lopes Batista, G.; Bertinetto, C. G.; Meurs, J.; Materić, D.; Bongers, C. C. W. G.; Allard, N. A. E.; Eijsvogels, T. M. H.; Holzinger, R.; Harren, F. J. M.; Jansen, J. J.; Hopman, M. T. E.; Cristescu, S. M. Exhaled Breath Reflects Prolonged Exercise and Statin Use during a Field Campaign. *Metabolites* **2021**, *11* (4), 192. https://doi.org/10.3390/metabo11040192.

(24) Schröder, D. Gaseous Rust: Thermochemistry of Neutral and Ionic Iron Oxides and Hydroxides in the Gas Phase. *The Journal of Physical Chemistry A* **2008**, *112* (50), 13215–13224. https://doi.org/10.1021/jp8030804.

(25) Fitzpatrick, E. M.; Ross, A. B.; Bates, J.; Andrews, G.; Jones, J. M.; Phylaktou, H.; Pourkashanian, M.; Williams, A. Emission of Oxygenated Species from the Combustion of Pine Wood and Its Relation to Soot Formation. *Process Safety and Environmental Protection* **2007**, *85* (5), 430–440. https://doi.org/10.1205/psep07020.

(26) Stockwell, C. E.; Veres, P. R.; Williams, J.; Yokelson, R. J. Characterization of Biomass Burning Emissions from Cooking Fires, Peat, Crop Residue, and Other Fuels with High-Resolution Proton-Transfer-Reaction Time-of-Flight Mass Spectrometry. *Atmospheric Chemistry and Physics* **2015**, *15* (2), 845–865. https://doi.org/10.5194/acp-15-845-2015.

(27) Tani, A.; Hayward, S.; Hewitt, C. N. Measurement of Monoterpenes and Related Compounds by Proton Transfer Reaction-Mass Spectrometry (PTR-MS). *International Journal of Mass Spectrometry* **2003**, *223–224*, 561–578. https://doi.org/10.1016/S1387-3806(02)00880-1.

(28) Kari, E.; Miettinen, P.; Yli-Pirilä, P.; Virtanen, A.; Faiola, C. L. PTR-ToF-MS Product Ion Distributions and Humidity-Dependence of Biogenic Volatile Organic Compounds. *International Journal of Mass Spectrometry* **2018**, *430*, 87–97. https://doi.org/10.1016/j.ijms.2018.05.003.
